# Activation of AKT via a dual mechanism enhances the susceptibility of melanoma cells to glucose deprivation

**DOI:** 10.1038/s41419-025-07906-4

**Published:** 2025-08-07

**Authors:** Guang-xi Yang, De-liang Peng, Lin Chen, Yu Qian, Le He, Xiao-yan Chen, Wen-bin Hong, Cai-ming Wu, Hang-zi Chen

**Affiliations:** The First Affiliated Hospital of Xiamen University, State Key Laboratory of Cellular Stress Biology, School of Life Sciences, Xiamen University, Xiamen, Fujian Province PR China

**Keywords:** Kinases, Cell death, Cancer metabolism, Phosphorylation

## Abstract

Protein kinase AKT plays a broad role in promoting energy production in nutrient-rich environments. However, its roles under metabolic stress remain elusive. Herein, we demonstrate a dual mechanism for AKT activation during glucose deprivation. On one hand, glucose deprivation leads to increased levels of ADP and NADP^+^, which directly bind to spleen tyrosine kinase (SYK) and induce a conformational alteration of SYK, resulting in self-activation. The activated SYK further triggers PI3K-dependent activation of AKT. On the other hand, elevated ROS upon glucose deprivation promotes oxidative dimerization of PDK1, thereby facilitating the recognition and activation of AKT. In melanoma cells, AKT plays a critical role in elevating ROS levels and inducing cell death during glucose deprivation. Overall, this study not only establishes a novel connection between energy insufficiency and AKT activation via a dual mechanism but also provides insights into the role of AKT in sensitizing cells to metabolic stress.

## Introduction

Alterations in glucose metabolism are one of the most prominent metabolic shifts that distinguish cancer cells from their normal cellular counterparts. This metabolic characteristic was initially described almost a century ago by Otto Warburg and is termed aerobic glycolysis or the Warburg effect [1]. Tumors primarily rely on glycolysis for energy production, and as tumors become more malignant, their energy demands increase significantly. Consequently, the tumor microenvironment often experiences a state of glucose deprivation [2]. However, emerging evidence suggests that cancer cells, including melanoma, can develop resistance to glucose deprivation through various mechanisms [3]. Therefore, modulating the adaptation of tumor cells to glucose deprivation has emerged as a promising strategy to restrict tumor development.

The protein kinase B (PKB/AKT) signaling pathway plays a crucial role in the growth of numerous tumor cells, regulating cellular survival, proliferation, growth, apoptosis, and glycogen metabolism [4]. The AKT family comprises three isoforms: AKT1, AKT2, and AKT3. During the tumorigenesis process, these three family members exhibit distinct yet overlapping roles [5]. Thr308 and Ser473 are two critical phosphorylation sites on the AKT protein that are essential for its activation [6]. Extracellular signals initiate a cascade that activates phosphoinositide 3-kinase (PI3K), which generates the second messenger phosphatidylinositol 3,4,5-trisphosphate (PIP3) to recruit AKT to the plasma membrane, and positioning it for activation through phosphorylation at its Ser473 and Thr308 residues, mediated respectively by mTORC2 and 3-phosphoinositide-dependent protein kinase 1(PDK1) [7, 8]. Numerous studies have demonstrated that AKT is closely associated with cellular energy metabolism and participates in the regulation of glucose metabolism [9].

The PI3K can be activated by a diverse range of regulatory factors, including GPCRs, RTKs, SYK, JAK, RAS, and others [10]. PIP3, the product of PI3K activation, not only recruits AKT to plasma membrane, but also binds to the PH domain of PDK1 to facilitate its recruitment to the plasma membrane for phosphorylation of AKT Thr308 residue and subsequent completion of its activation. It has been reported that phosphorylation of serine 241 in the activation loop of PDK1 is an essential element required for its kinase activity, catalyzed by itself but not other kinases [11]. In addition to this primary phosphorylation event, other phosphorylation events [12], as well as dimerization, are also involved in the activation of PDK1 [13].

Spleen tyrosine kinase (SYK) is a 72 kDa non-receptor tyrosine kinase that plays an essential role in immunity and a variety of tumors [14, 15]. In response to extracellular stimuli, the SH2 domain of SYK binds to the immunoreceptor tyrosine-based activation motif (ITAM) domain within the intracellular region of BCR or TCR [16]. This interaction triggers the activation of SYK kinase, which can be facilitated by the src family of kinases or through autophosphorylation [17], subsequently facilitating the activation of PI3K and the accumulation of PIP3 [18]. Studies have demonstrated SYK kinase plays a pivotal role as a growth suppressor in melanoma [19]. Furthermore, the viability of melanoma cells is enhanced when SYK kinase is inhibited under oxidative metabolic stress conditions [20].

In this study, we found that AKT is activated through the PI3K-PIP3 pathway in various tumor cell lines (melanoma, hepatoma, colon cancer, and breast cancer) under glucose deprivation conditions. By screening for inhibitors that regulate PI3K, we provided evidence that inhibiting SYK effectively blocks the activation of AKT induced by glucose deficiency. Interestingly, SYK activation occurs through binding of ADP or NADP^+^ to the SH2 domain of SYK under glucose deprivation - a novel mechanism not previously reported. Additionally, the oxidative dimerization of PDK1 promotes the recognition of AKT by PDK1, further promoting the phosphorylation of Thr308 on AKT. This glucose deprivation-induced AKT activation is closely associated with elevated ROS and melanoma cell death.

## Results

### Glucose deprivation induces the activation of AKT

**Fig. 1:**
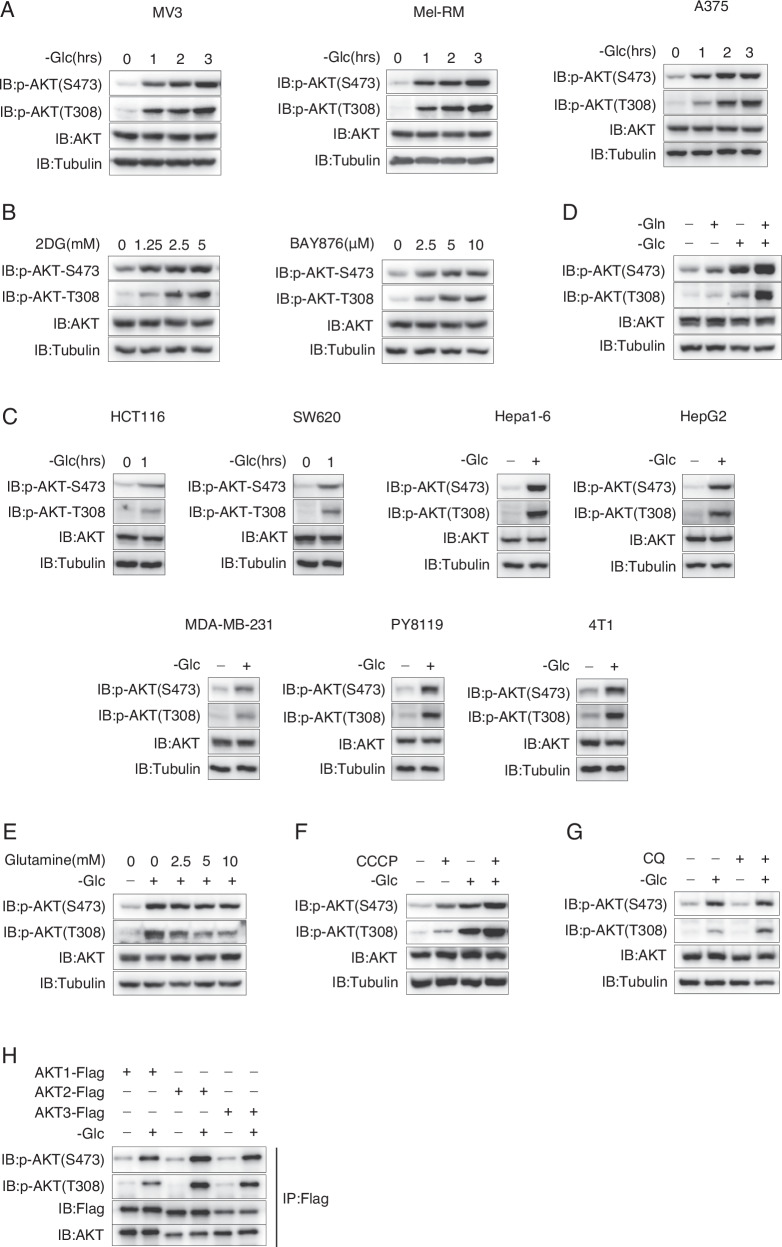
Energy deficiency induces activation of AKT in various tumor cell lines. **A** Glucose deprivation induces AKT phosphorylation. Melanoma cell lines, include MV3, Mel-RM, and A375, was cultured under glucose starvation conditions for the time points as indicated, and AKT phosphorylation levels at Ser473 and Thr308 were determined. **B** 2DG or BAY876 induces AKT activation. A375 cells was treated with 2DG or BAY876 at the concentrations as indicated for 3 h. AKT phosphorylation levels at Ser473 and Thr308 were determined. **C** Glucose deprivation induces AKT phosphorylation in many tumor cell lines. Human colon cancer cell lines HCT116 and SW620, human HCC cell line HepG2, mouse HCC cell line Hepa1-6, human breast cancer cell line MDA-MB-231, and murine mammary cancer cell lines PY8119 and 4T1 were cultured under glucose starvation for 1 h. AKT phosphorylation levels at Ser473 and Thr308 were determined. **D** Glutamine deprivation upregulates AKT phosphorylation. A375 cells were cultured in media with or without glucose or glutamine as indicated for 3 h. AKT phosphorylation levels at Ser473 and Thr308 were determined. **E** Glutamine impairs glucose deprivation-induced AKT phosphorylation. A375 cells were cultured in a glucose-free medium supplemented with varying concentrations of glutamine as indicated for 3 h, and AKT phosphorylation levels at Ser473 and Thr308 were determined. **F**, **G** CCCP or CQ upregulates AKT phosphorylation. A375 cells were treated with CCCP (2.5 μM, **F**) or CQ (20 μM, **G**) under control or glucose starvation conditions for 3 h. AKT phosphorylation was determined. **H** All AKT family members are activated upon glucose deprivation. Flag tagged AKT1-3 were transfected into A375 cells, AKTs were then immunoprecipitated from control or glucose deprived-A375 cells using anti-Flag antibody. The phosphorylation of AKTs was detected in the immunoprecipitants.

AKT plays a broad role in promoting energy production in nutrient-rich environments [21], but its function under metabolic stress remains controversial. Interestingly, we unexpectedly observed a gradual increase in the phosphorylation of AKT at Thr308 and Ser473 with prolonged glucose deprivation in MV3, Mel-RM, and A375 human melanoma cells (Fig. 1A), indicating the activation of AKT during glucose deprivation. To simulate glucose deprivation conditions, we treated cells with 2-deoxy-D-glucose (2DG), a non-metabolizable glucose analog [22], and found that 2DG could also induce the phosphorylation of AKT in a dose dependent manner (Fig. 1B, left). Glucose transporters (Gluts) are responsible for glucose uptake. Inhibition of Gluts activity using BAY-876 also resulted in dose-dependent activation of AKT (Fig. 1B, right). These findings suggest that AKT can be activated under metabolic stress caused by glucose deprivation in melanoma cells. Importantly, we also detected glucose deprivation-induced activation of AKT in HCT116 and SW620 human colon cancer cells, HepG2 human hepatocellular carcinoma (HCC) cells, Hepa 1-6 mouse HCC cells, MDA-MB-231 human breast cancer cells, and PY8119 and 4T1 murine mammary cancer cells (Fig. 1C). Therefore, it appears that glucose deprivation may have a general effect on activating AKT across various cancer cell lines.

To assess the potential activation of AKT by alternative forms of metabolic stress, A375 cells were cultured under glutamine-deprived conditions. While the increase in AKT phosphorylation due to glutamine deprivation was modest, a synergistic activation of AKT was observed when both glucose and glutamine were deprived (Fig. 1D). Conversely, supplementation of glutamine in glucose-deprived cells dose-dependently impaired AKT phosphorylation (Fig. 1E). Furthermore, treatment of A375 cells with CCCP, a mitochondrial uncoupler that disrupts energy production dependent on mitochondrial respiration, or CQ, an autophagy inhibitor that abolishes autophagy-dependent energy production, significantly elevated AKT phosphorylation under glucose deprivation conditions (Fig. 1F, G). Henceforth, it can be inferred that AKT is activated during states of energy deficiency.

The AKT family consists of three members, namely AKT1, AKT2, and AKT3. To assess the specific activation of certain AKT members under glucose deprivation conditions, A375 cells were individually transfected with AKT1, AKT2, and AKT3. The findings demonstrated that glucose deprivation could induce comparable phosphorylation of all the AKTs (Fig. 1H), suggesting that all members of the AKT family can be activated in response to glucose deprivation. Therefore, it is plausible that glucose deprivation may activate the upstream activator of AKT.

### SYK is required for glucose deprivation-induced AKT activation

**Fig. 2:**
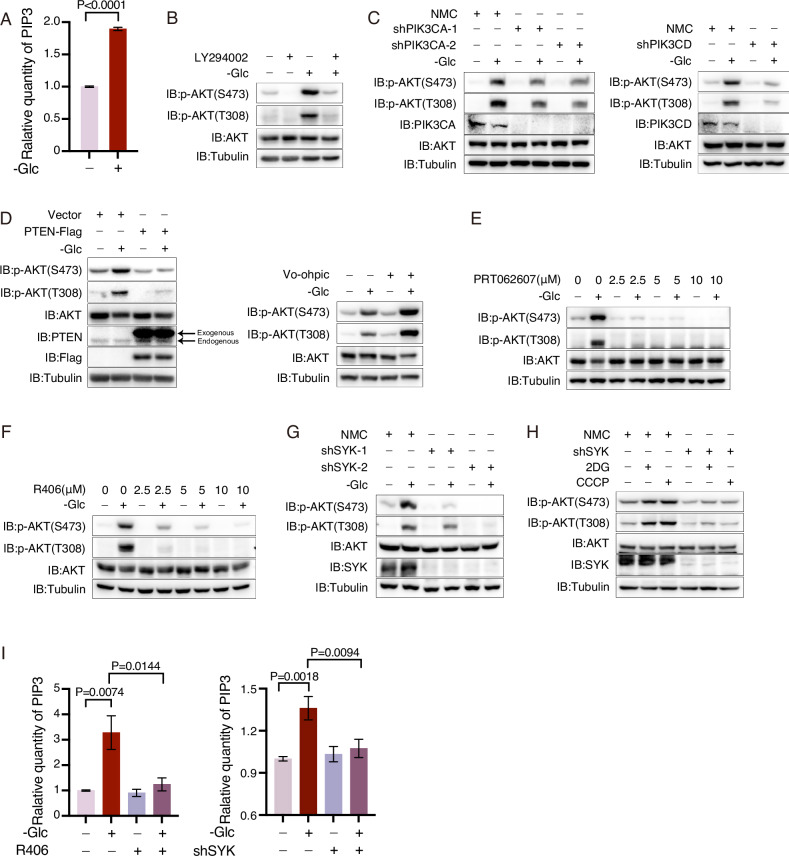
SYK is required for glucose deprivation induced AKT activation. **A** Glucose starvation elevates the level of PIP3. A375 cells were cultured in glucose-free medium for 2 h to detect the amount of PIP3 by ELISA. The data are presented as the means ± SEM of at least three independent experiments. **B**, **C** PI3K is crucial for glucose starvation-induced AKT phosphorylation. A375 cells were subjected to glucose deprivation for 3 h in the presence or absence of LY294002 (10 μM), a PI3K inhibitor (**B**). PIK3CA or PIK3CD was knocked down in A375 cells, subsequently, the cells were starved of glucose for 3 h (**C**). The phosphorylation status of AKT was assessed. **D** PTEN inhibits AKT activation induced by glucose starvation. The A375 cells overexpressing PTEN or control were cultured under glucose deprivation for 3 h (left). The A375 cells were treated with the PTEN inhibitor Vo-Ohipc (5 μM) while being cultured under glucose starvation for 3 h (right), and the phosphorylation of AKT was determined. **E**–**G** SYK is essential for glucose deprivation-induced AKT activation. The A375 cells were treated with SYK inhibitors PRT062607 (**E**) or R406 (**F**) as the indicated concentrations when cultured in glucose-free medium for 3 h, and the phosphorylation of AKT was assessed. The knockdown of SYK was achieved using two individual shRNAs in A375 cells (**G**), followed by culturing under glucose deprivation for 3 h to assess the phosphorylation status of AKT. **H** SYK is critical for AKT activation induced by energy deficiency. The SYK knockdown A375 cells were subjected to glucose starvation for 3 h in the presence of 2DG (2.5 mM) or CCCP (2.5 μM). The phosphorylation of AKT was subsequently assessed. **I** SYK is required for PIP3 upregulation induced by glucose deprivation. The A375 cells were treated with R406 (5 μM, left) or knocked out of SYK (right) and cultured in glucose-free medium for 2 h to quantify the level of PIP3. The data are presented as the means ± SEM of at least three independent experiments.

Phosphoinositide 3-kinase (PI3K) plays an essential role in AKT activation by catalyzing the synthesis of PIP3 [23]. Considering that glucose deprivation markedly increased cellular levels of PIP3 (Fig. 2A), we supposed that PI3K may play a role in glucose deprivation-induced AKT activation. Treatment of A375 cells with LY294002, a specific inhibitor for PI3K, almost completely abrogated the phosphorylation of AKT at Thr308 and Ser473 induced by glucose deprivation (Fig. 2B). Similarly, knockdown of *PIK3CA* or *PIK3CD* (the two catalytic subunits of PI3K) exhibited significantly diminished AKT activation under glucose deprivation conditions (Fig. 2C), suggesting that PI3K is indispensable for AKT activation upon glucose deprivation. PTEN, a phosphatase responsible for dephosphorylating PIP3, acts as a negative regulator of AKT [24]. Overexpression of PTEN almost completely abolished the induction of AKT phosphorylation by glucose deprivation (Fig. 2D, left). Conversely, inhibition of PTEN using its specific inhibitor VO-Ohpic substantially enhanced AKT phosphorylation during glucose deprivation conditions (Fig. 2D, right). Importantly, treatment with VO-Ohpic did not abolish the impact of glucose deprivation on AKT phosphorylation (Fig. 2D, right), thereby excluding the regulation of PTEN as a mechanism for AKT activation during glucose deprivation. Hence, it is plausible that glucose deprivation may stimulate the activation of PI3K, which generates PIP3 for subsequent AKT activation.

The involvement of PKA [25], Gβγ [26], IRS-1 [27], JAK [28], Ras [29], TGFβ receptor [30], calcium [31], and SYK [32] in PI3K activation has been reported. However, individual inhibition of these proteins using their respective inhibitors (H89 for PKA inhibition, Gallein for Gβγ inhibition, NT157 for IRS-1/2 inhibition, Pyridone for JAK inhibition, Lonafarnib for Ras inhibition, SB505124 for TGFβ receptor inhibition, and Ca^2+^ chelator BAPTA-AM did not abolish AKT phosphorylation induced by glucose deprivation (Fig. S1A). Interestingly, though, treatment with either PRT062607 or R406—both SYK inhibitors— significantly impaired glucose deprivation-induced AKT phosphorylation in a dose-dependent manner (Fig.2E, F). Furthermore, knockdown of SYK also abolished AKT phosphorylation induced by glucose deprivation, as well as 2DG or CCCP treatment (Fig. 2G, H). Combined with the fact that pharmacological inhibition or knockdown of SYK abolished glucose deprivation-induced PIP3 elevation (Fig. 2I), it could be concluded that SYK plays a crucial role as an upstream activator of PI3K, promoting AKT activation during glucose deprivation.

To determine whether SYK can activate all members of the AKT family, Flag-tagged AKT1, AKT2, and AKT3 were transfected into control or SYK-knockdown A375 cells. Subsequently, the phosphorylation levels of AKTs under glucose starvation conditions were detected following immunoprecipitation of Flag-AKTs. The results demonstrated that SYK knockdown nearly abolished glucose starvation-induced phosphorylation of AKT2 and AKT3, while it only partially impaired AKT1 phosphorylation (Fig. S1B). Therefore, these findings suggest that although SYK plays a critical role in the activation of AKT1, AKT2, and AKT3, alternative mechanisms may also contribute to AKT1 activation under glucose starvation conditions.

### Glucose deprivation enhances SYK activity

**Fig. 3:**
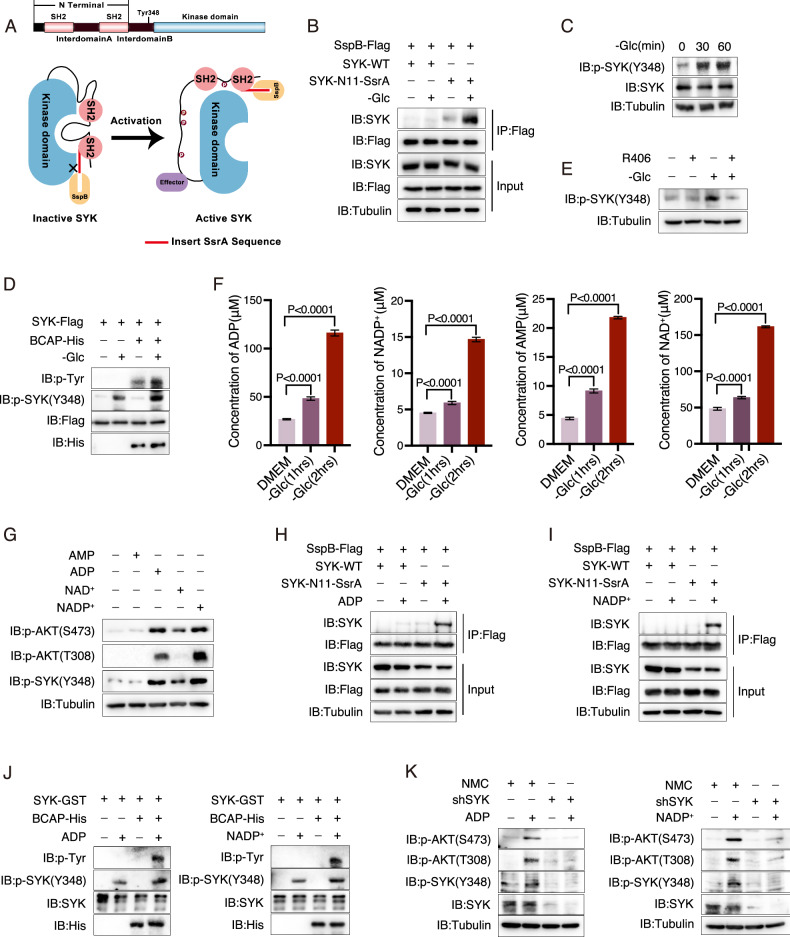
Glucose starvation elevates SYK activity. **A** A schematic diagram illustrating the “binder/tag” methodology. The SsrA sequence (AANDENY) was inserted after Asn11 of SYK, which is enclosed within the SYK protein in an inactive state. The exposure of SsrA to the external environment upon activation facilitates its interaction with SspB. **B** Glucose deprivation results in conformation alteration of SYK. The A375 cells overexpressing SspB-Flag were transfected with SYK-WT or SYK-SsrA (N11) mutant, respectively. After glucose starvation for 1 h, Flag immunoprecipitation was performed to determine the conformational change of SYK. **C**, **D** Glucose deprivation activates SYK. The A375 cells were cultured in a glucose-free medium for 30 or 60 min, and the phosphorylation at Tyr348 of SYK was assessed (**C**). A375 cells overexpressing SYK-Flag were cultured in a glucose-free medium for 1 h. Immunoprecipitation of SYK from control or glucose-deprived A375 cells was performed, followed by incubation with bacterially expressed BCAP to determine tyrosine phosphorylation (**D**). **E** R406 inhibits SYK activation induced by glucose starvation. The A375 cells were subjected to treatment with R406 (5 μM) under conditions of glucose starvation. The phosphorylation at the Tyr348 of SYK was assessed. **F** Glucose starvation elevates the intracellular levels of ADP, NADP^+^, AMP, and NAD^+^ in A375 cells. Mass spectrometry was employed to determine the quantities of ADP, NADP^+^, AMP, and NAD^+^. Cell volume was calculated by averaging measurements from at least 50 cells using Imaris. The data are represented as the means ± SEM of at least three independent experiments. **G** ADP and NADP+ activate SYK and AKT. The A375 cells were pre-treated with SLO (50 μg/μl) for 10 min, followed by treatment with AMP (200 μM), ADP (200 μM), NAD^+^ (200 μM), or NADP^+^ (200 μM) for 40 min without SLO. The phosphorylation of SYK and AKT was assessed. **H**, **I** ADP, and NADP^+^ alter conformation of SYK. The A375 cells overexpressing SspB-Flag were transfected with either SYK-WT or the SYK-SsrA (N11) mutant, respectively. Prior treatment, both cell lines were pretreated with SLO (50 μg/μl) for 10 min, followed by ADP (200 μM, **H**) or NADP^+^ (200 μM, **I**) treatment for 40 min without SLO. The protein level of SYK was determined in the anti-Flag immunoprecipitants. **J** ADP and NADP^+^ activate SYK. The bacterially expressed SYK and BCAP were incubated with or without ADP (200 μM, left) or NADP^+^ (200 μM, right) for 1 h in an in vitro kinase buffer at 30 °C. The reaction was stopped by adding loading buffer. The phosphorylation of tyrosine was subsequently determined. **K** SYK is required for AKT activation induced by ADP or NADP^+^. The Control or SYK-KD A375 cells were pre-treated with SLO (50 μg/μl) for 10 min, followed by ADP (200 μM, left) or NADP^+^ (200 μM, right) treatment for 40 min without SLO. The phosphorylation of AKT was assessed.

We then investigated whether glucose deprivation could trigger the activation of SYK. In its inactive state, SYK is autoinhibited by the binding of N-terminal domain to the kinase domain. Disruption of this autoinhibition state leads to autophosphorylation at the interdomain, which sustains SYK activation [33, 34]. To examine if glucose deprivation induced a conformational change in SYK that disrupted its autoinhibition state, we employed a previously reported “binder/tag” approach [35]. In this system, a 7-residue peptide SsrA (the tag) was inserted into the SH2 domain of SYK, and SspB (the binder), an 18 kDa protein with high affinity for SsrA, was used to pull down SYK when the autoinhibition of SYK was disrupted and exposed the SsrA peptide (Fig. 3A). Using this system, we observed that glucose deprivation significantly increased exposure of the inserted SsrA peptide, indicating a conformational change in SYK (Fig. 3B). This conformational alteration is closely associated with the phosphorylation of Tyr348 at the interdomain in SYK (Fig. 3C). When SYK was immunoprecipitated from glucose starved cells and subsequently incubated with BCAP, a substrate protein for SYK [36], in vitro, the phosphorylation of BCAP could be clearly detected (Fig. 3D). These results indicated that glucose deprivation could induce the conformational alteration and subsequent activation of SYK.

The involvement of Src in SYK phosphorylation and activation was previously reported [14]; however, inhibition of Src by its inhibitor did not impair glucose deprivation-induced SYK phosphorylation and AKT activation (Fig. S2A), thus ruling out the role of Src in SYK activation during glucose deprivation. It is believed that the recruitment of SYK to the plasma membrane is crucial for its activation [37]; however, both the level of SYK and its phosphorylation at Tyr348 in the plasma membrane fraction remained unchanged upon glucose deprivation (Fig. S2B). The fact that glucose deprivation-induced SYK Tyr348 phosphorylation could be abolished by R406, a specific inhibitor for SYK (Fig. 3E), suggested that glucose deprivation may induce conformational changes in SYK to facilitate its autophosphorylation and activation.

Given the activation of AKT in various metabolic stress conditions, we postulated that the metabolites enriched during metabolic stress might dictate SYK activation. The concentrations of AMP, ADP, NAD^+^, and NADP^+^, which serve as indicators of cellular energy and reductive capacity, were significantly elevated under glucose-deprived conditions (Fig. 3F). This suggests a deficiency in the cell’s energy and reducing power when glucose is unavailable. Subsequently, we investigated whether these metabolites could regulate SYK and AKT activation. To facilitate their entry into cells during incubation experiments, A375 cells were permeabilized using streptolysin O (SLO). Our findings revealed that ADP and NADP^+^, but not AMP or NAD^+^, markedly increased phosphorylation levels of SYK Tyr348, consequently leading to AKT phosphorylation (Fig. 3G). The utilization of the “binder/tag” approach revealed a significant conformational change in SYK upon stimulation with ADP and NADP^+^ (Fig. 3H, I). In an in vitro kinase activity assay, incubation of bacterially expressed SYK with ADP or NADP^+^ significantly enhanced its ability to phosphorylate BCAP (Fig. 3J), thereby confirming the direct activation of SYK by ADP or NADP^+^. Notably, when SYK was knocked down, both ADP and NADP^+^ lost their capacity to activate AKT (Fig. 3K). Collectively, it can be concluded that under glucose deprivation conditions, elevated levels of ADP and NADP^+^ activate SYK, which subsequently induces AKT activation.

### ADP and NADP^+^ binds to SYK to promote SYK activation

**Fig. 4:**
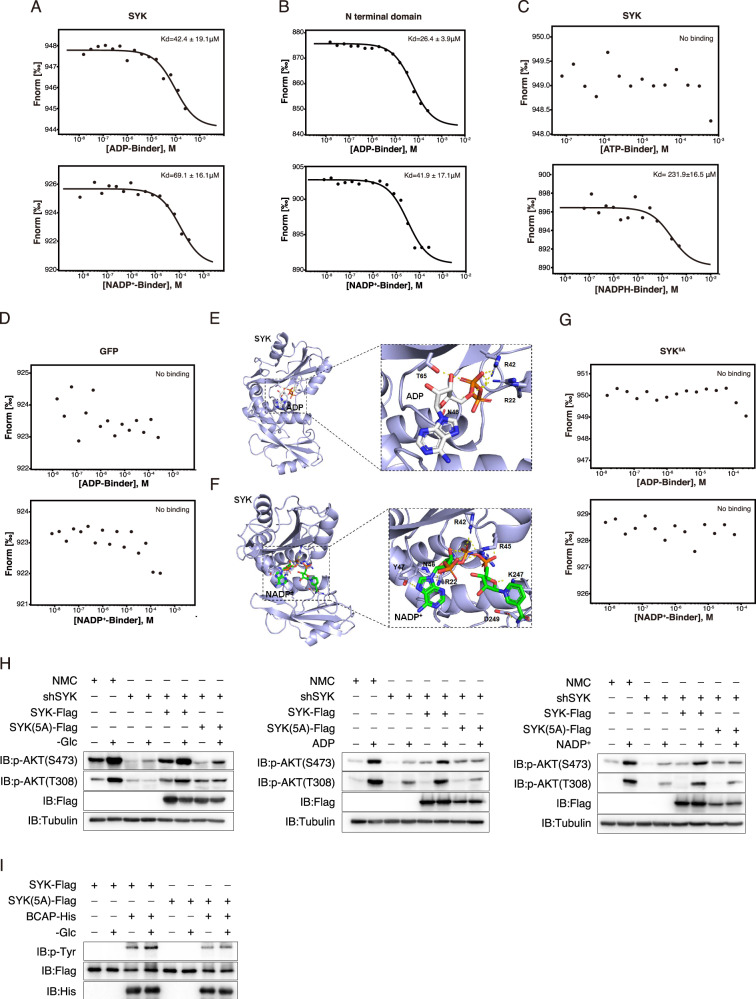
NADP^+^ and ADP bind to SYK to induce its activation. **A**-**D** ADP and NADP^+^ bind to SYK. Representative binding curves of full-length SYK (**A**), N-terminal of SYK (**B**) or GFP (**D**) with ADP or NADP^+^, or SYK with ATP or NADPH (**C**) were obtained using MST in three independent experiments. The Kd values were determined and presented. **E**, **F** ADP **(E**) and NADP^+^ (**F**) interact with SYK, respectively. ADP, NADP^+^, and interacting residues are shown in rainbow sticks, hydrogen bonds are in yellow dashed lines, and cyan cartoon indicates the tandem SH2 domains of SYK (PDB: 7Q5W). Residue interactions were analyzed using PyMOL. **G** ADP or NADP^+^ does not bind to SYK^5A^. Representative binding curves of SYK^5A^ with ADP or NADP^+^ were obtained using MST in three independent experiments. **H** The binding of SYK with ADP or NADP^+^ is critical for glucose starvation-induced AKT activation. The SYK knockdown A375 cells were separately transfected with SYK or its mutant SYK^5A^. Subsequently, the cells were cultured in a glucose-free medium for 3 h (left), treated with ADP (200 μM, middle) or NADP^+^ (200 μM, right) for 40 min to assess the phosphorylation of AKT. **I** SYK^5A^ retains its activity in phosphorylating its substrate. A375 cells were transfected with SYK or its mutant SYK^5A^. SYK and SYK^5A^ from control or glucose-deprived (3 h) cells were immunoprecipitated and subsequently incubated with bacterially expressed BCAP in an in vitro kinase buffer. The phosphorylation of tyrosine was subsequently determined.

The above results demonstrate that ADP and NADP^+^ can induce the conformational change of SYK and directly activate SYK in vitro, suggesting a potential direct binding between ADP/NADP^+^ and SYK. To investigate this further, we employed microscale thermophoresis (MST) to determine the direct interaction between ADP or NADP^+^ with SYK. Our findings reveal that ADP binds to both SYK (with a Kd of 42.4 ± 19.1 μM) and the N-terminal domain of SYK (with a Kd of 26.4 ± 3.9 μM), while NADP^+^ exhibits affinity towards SYK (with a Kd of 69.1 ± 16.1 μM) as well as the N-terminal domain of SYK (with a Kd of 41.9 ± 17.1 μM) (Fig. 4A, B). No significant affinity was observed between ATP and SYK, and the binding affinity of NADPH with SYK (Kd = 231.9 ± 16.5 μM) was considerably weaker compared to that of NADP^+^ with SYK (Fig. 4C). Furthermore, there is no apparent affinity observed between NADP^+^ or ADP with GFP protein (Fig. 4D). Hence, these results confirmed the direct interaction between ADP/NADP^+^ with the N-terminal domain of SYK protein.

We subsequently conducted molecular docking simulations to simulate the interaction between ADP and NADP^+^ with the N-terminal domain of the SYK protein. Illustrations of the ADP/SYK and NADP^+^/SYK complexes were generated, and residue interactions were analyzed using PyMOL. Specifically, ADP and NADP^+^ bind to a common pocket on SYK, primarily engaging in hydrogen bond interactions with polar amino acid side chains of SYK (Fig. 4E, F). MST assays further demonstrated that mutation of key residues (Arg22, Arg42, Arg45, Gln46, and Lys247) within the N-terminal domain of SYK into Ala (termed as SYK^5A^) significantly diminished its interaction with ADP and NADP^+^ (Fig. 4G). Consequently, in cells expressing SYK^5A^ mutant protein, both glucose deprivation-induced AKT phosphorylation as well as the effects of ADP and NADP^+^ on AKT activation were substantially attenuated (Fig. 4H). Notably, when SYK and SYK^5A^ were immunoprecipitated from control or glucose-deprived A375 cells and subsequently incubated with bacterially expressed BCAP in vitro, SYK^5A^ could phosphorylate BCAP as effectively as SYK, suggesting that SYK^5A^ retained its activity in phosphorylating its substrate. However, glucose deprivation significantly enhanced SYK-mediated BCAP phosphorylation but did not affect SYK^5A^-mediated phosphorylation (Fig. 4I). Collectively, these findings suggest that under conditions of glucose deprivation, SYK may serve as a sensor for ADP and NADP^+^, leading to the activation of PI3K-AKT.

### Glucose deprivation-activated AKT3 elevates ROS to facilitate cell death

**Fig. 5:**
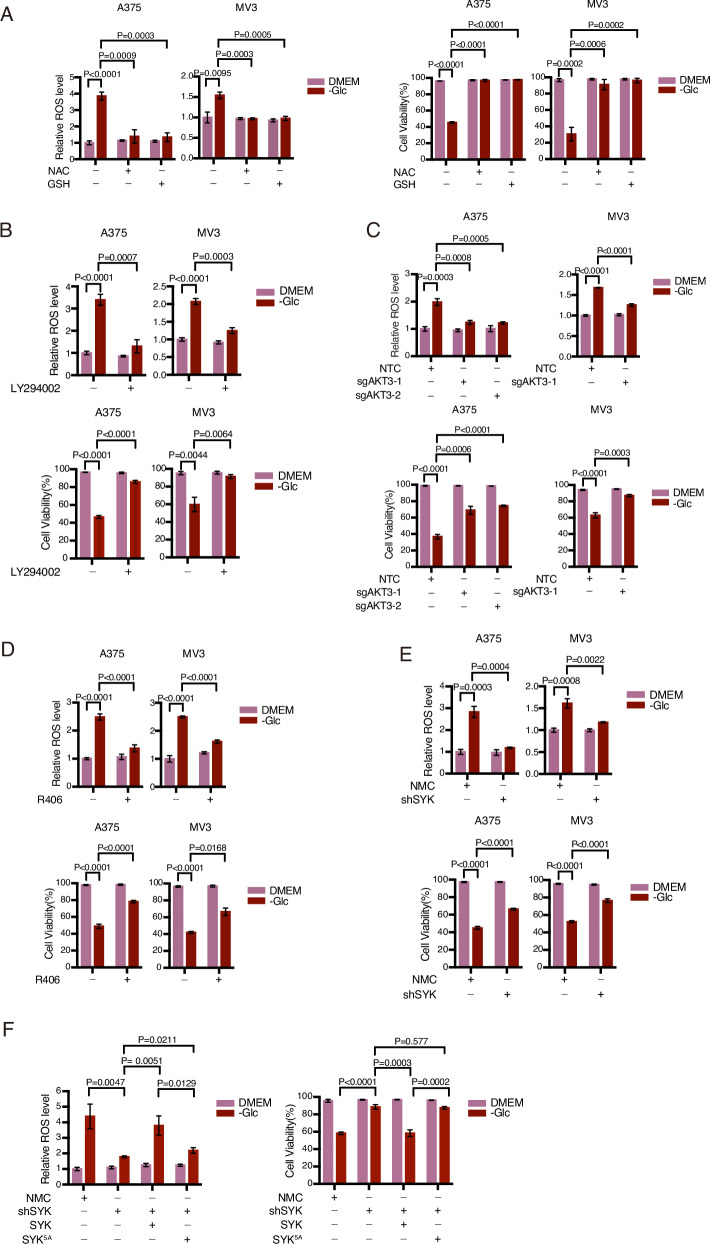
Activation of AKT elevate ROS level to promote glucose starvation induced cell death. **A** NAC and GSH reduce ROS levels and cell death upon glucose deprivation. The A375 or MV3 cells were subjected to glucose starvation for 6 h in the presence of NAC (5 mM) or GSH (2.5 mM) to assess the level of ROS. The cell viability was evaluated after 9 h. **B** PI3K is required for glucose starvation-induced ROS elevation and cell death. The A375 or MV3 cells were subjected to glucose starvation in the presence of LY294002 (10 μM) to assess the level of ROS (6 h) or cell viability (9 h). **C** AKT3 is required for ROS elevation and cell death induced by glucose deprivation. The A375 or MV3 cells with AKT3 knockout were cultured in a glucose-free medium for 6 h to measure ROS level, and for 9 h to assess cell viability. **D**, **E** SYK is crucial for glucose starvation-induced ROS elevation and cell death. The A375 or MV3 cells were treated with R406 (5 μM) and cultured in a glucose-free medium for 6 h to measure ROS levels, and for 9 h to assess cell viability (**D**). A375 or MV3 cells with SYK knockdown were cultured in a glucose-free medium, and both ROS level and cell viability were analyzed (**E**). **F** SYK^5A^ impairs glucose starvation-induced ROS and cell death. The SYK-KD A375 cells were transfected separately with SYK or SYK^5A^. The cells were then subjected to glucose starvation for 6 h to measure the level of ROS, and for 9 h to assess cell viability. The data are presented as the means ± SEM of three independent experiments.

We subsequently investigated the biological implications of AKT activation induced by glucose deprivation. It has been reported that glucose deprivation induces an increase in reactive oxygen species (ROS) levels, leading to melanoma cell death [38]. Indeed, we observed a clear elevation of ROS upon glucose deprivation in both A375 and MV3 cells, which was closely associated with cellular demise. The restoration of cell viability through the inhibition of ROS elevation using NAC or GSH (Fig. 5A) confirmed the role of ROS in glucose deprivation-induced cell death. Furthermore, attenuation of both ROS levels and cell death induced by glucose deprivation was achieved through the inhibition of AKT activation using LY294002 (Fig. 5B). qPCR analysis revealed that AKT3 was the predominant member expressed in A375 cells (Fig. S3A). Only knockout of AKT3, but not AKT1 or AKT2, abolished glucose deprivation-induced phosphorylation of AKT (Fig. S3B–D), suggesting that the activation of AKT3 may play a role in response to glucose deprivation. Indeed, knockout of AKT3, rather than AKT1 or AKT2, impaired glucose deprivation-induced ROS levels and protected A375 and MV3 cells from cell death caused by glucose deprivation (Figs. 5C and S3E, F). Therefore, AKT3 is implicated in mediating cell death under conditions of glucose deprivation.

Inhibition of SYK activity by R406 or knockdown of SYK also attenuated ROS elevation and cell death under conditions of glucose deprivation (Fig. 5D, E). Importantly, in SYK knockdown cells, transfection with wildtype SYK restored the level of ROS and cell death induced by glucose deprivation, whereas transfection with mutant SYK^5A^ did not have this effect (Fig. 5F). Collectively, these results indicate that the activation of AKT3 mediated by SYK is crucial for the elevation of ROS and induction of cell death upon glucose deprivation in melanoma cells.

### ROS facilitate AKT activation by inducing PDK1 dimerization and activation

**Fig. 6:**
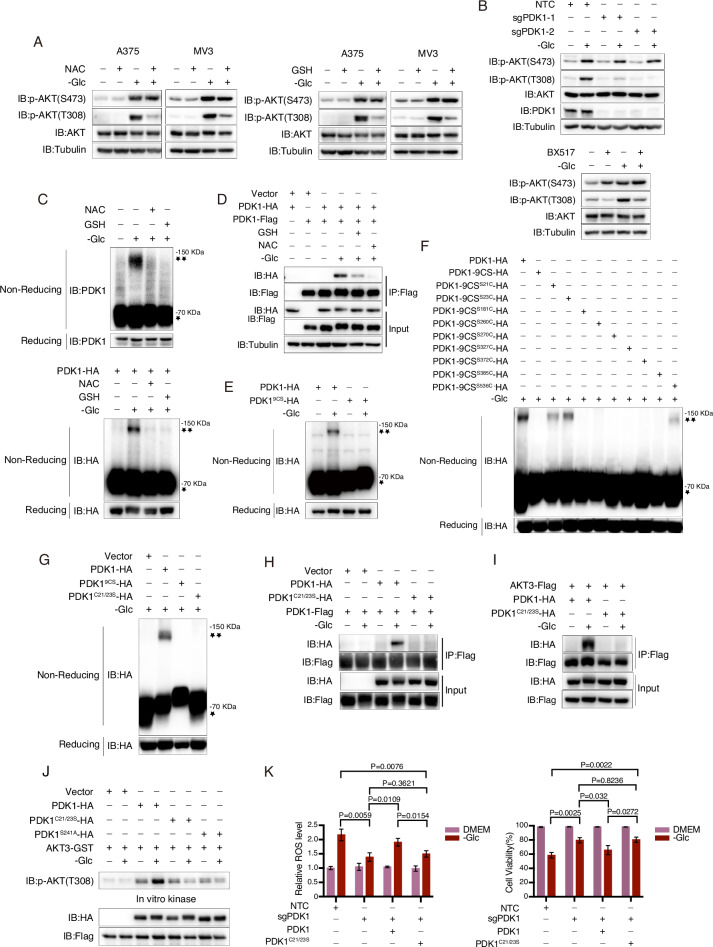
Glucose starvation promotes the dimerization and activation of PDK1. **A** NAC and GSH impair glucose starvation-induced AKT Thr308 phosphorylation. The A375 or MV3 cells were subjected to glucose starvation for 3 h in the presence of NAC (5 mM, left) or GSH (2.5 mM, right). The phosphorylation of AKT at Ser473 or Thr308 sites was assessed. **B** PDK1 is responsible for the phosphorylation of AKT Thr308. The A375 cells with PDK1 knockout (top) or treated with BX517 (5 μM, bottom) were cultured in a glucose-free medium for 3 h to assess the phosphorylation of AKT at Ser473 or Thr308 sites. **C**, **D** Glucose starvation induces oxidative dimerization of PDK1. The A375 cells or the A375 cell line overexpressing PDK1 were cultured in a glucose-free medium for 3 h in the presence of NAC (5 mM) or GSH (2.5 mM). The dimerization of endogenous (**C**, top) or exogenous (**C**, bottom) PDK1 was determined in non-reducing conditions or via co-immunoprecipitation (**D**). *, PDK1 monomer; **, PDK1 dimer. **E** Cysteine residues of PDK1 are critical for the oxidative dimerization of PDK1. The HA-tagged PDK1 or its mutant PDK1^9CS^ were transfected separately into A375 cells, and the oxidative dimerization of PDK1 or PDK1^9CS^ induced by glucose deprivation was determined under non-reducing conditions. **F** Cys21 and Cys23 in PDK1 are responsible for glucose deprivation-induced PDK1 dimerization. The dimerization of single-point PDK1 rescue mutants based on PDK1^9CS^ was observed in A375 cells subjected to glucose starvation for 3 h. **G**, **H** PDK1^C21/23S^ does not undergo dimerization in response to glucose deprivation. The dimerization of PDK1 and PDK1^C21/23S^ was determined in non-reducing conditions (**G**) or via co-immunoprecipitation (**H**) upon glucose starvation for 3 h. **I** PDK1^C21/23S^ fails to bind to AKT3. The interaction of PDK1 or PDK1^C21/23S^ with AKT3 was determined by co-immunoprecipitation. **J** PDK1^C21/23S^ cannot phosphorylate AKT3 in vitro. The bacterially expressed AKT3 was incubated with PDK1, PDK1^C21/23S^, or PDK1^S241A^ (negative control) from A375 cells treated with or without glucose deprivation. The phosphorylation of AKT Thr308 was determined. **K** Cys21 and Cys23 in PDK1 are vital for glucose deprivation-induced ROS and cell death. The PDK1-KD A375 cells were separately transfected with PDK1 or PDK1^C21/23S^. The cells were then cultured under glucose starvation conditions for 6 h to measure ROS level (left), and for 9 h to assess cell viability (right). The data are presented as the means ± SEM of three independent experiments.

Considering the role of AKT in glucose deprivation-induced ROS elevation, we investigated whether ROS levels are involved in the regulation of AKT. To address this, A375 and MV3 cells were subjected to glucose deprivation in the presence of NAC or GSH. Interestingly, while NAC and GSH had no impact on the phosphorylation level of AKT at Ser473, they significantly attenuated the phosphorylation of AKT Thr308 during glucose deprivation (Fig. 6A). PDK1 plays a crucial role in mediating AKT Thr308 phosphorylation [39]. Notably, when PDK1 was knocked out or inhibited by BX517, the glucose deprivation-induced phosphorylation of AKT Thr308 was completely abolished (Fig. 6B). Hence, it is plausible that glucose deprivation-induced ROS may be involved in regulating AKT Thr308 phosphorylation through PDK1.

Since protein oxidative oligomerization occurs upon elevated levels of ROS [40], we investigated whether PDK1 undergoes oxidative oligomerization during glucose deprivation. As anticipated, glucose deprivation clearly induced the dimerization of both endogenous and exogenous PDK1, as observed in non-reducing gels (Fig. 6C). Consistently, co-immunoprecipitation assays also demonstrated that glucose deprivation significantly enhanced the interaction between PDK1-HA and PDK1-Flag (Fig. 6D). This dimerization was effectively inhibited by treatment with GSH or NAC (Fig. 6C, D), indicating the ROS-dependent dimerization i.e., oxidative dimerization of PDK1. Cysteine residues play a critical role in oxidative dimerization of proteins. There are nine cysteine residues present in PDK1, and when all these cysteines were mutated to serine (PDK1^9CS^), the glucose deprivation-induced dimerization of PDK1 was completely abolished (Fig. 6E). Subsequently, individual rescue of the Cys residues was performed based on the 9CS mutant, and revealed that glucose deprivation-induced dimer formation was observed at Cys21 and Cys23 (Fig. 6F). Simultaneous mutation of these two cysteine residues abrogated the glucose deprivation-induced dimerization of PDK1, as evidenced in non-reducing gels and co-immunoprecipitation assays (Fig. 6G, H). Therefore, it can be concluded that ROS generated during glucose deprivation promote the Cys21- and Cys23-dependent dimer formation of PDK1.

We then investigated the role of PDK1 dimerization in AKT activation. It was clearly demonstrated that the interaction between PDK1 and AKT3 was significantly enhanced under conditions of glucose deprivation. However, when Cys21 and 23 were mutated, the effect of glucose deprivation on the PDK1-AKT3 interaction was dramatically impaired (Fig. 6I). The in vitro kinase assay revealed that immunoprecipitated PDK1 from glucose-starved cells effectively induced phosphorylation of AKT3 detected by anti-phospho-Thr308 antibody. Conversely, the ability of the PDK1^C21/23S^ mutant to phosphorylate AKT3 was lost. As a negative control, it was observed that PDK1^S241A^, a kinase-dead mutant of PDK1, also failed to phosphorylate AKT3 (Fig. 6J). Consequently, in A375 cells expressing PDK1^C21/23S^ mutation, both ROS elevation and cell death induced by glucose deprivation were greatly diminished (Fig. 6K). In conclusion, these findings suggest that the AKT3-dependent increase in ROS during glucose deprivation acts as an activator of PDK1, thereby further activating AKT through a feedforward mechanism.

The aforementioned data indicate that, under glucose-deprived conditions, AKT is activated through a dual mechanism: ADP/NADP^+^-mediated activation of the SYK-PI3K axis and ROS-mediated activation of PDK1. We further investigated which mechanism predominates. It has been established that phosphorylation of AKT at both Ser473 and Thr308 is essential for its complete activation [6]. Knockdown of SYK abolished AKT phosphorylation at both Ser473 and Thr308, whereas knockdown of PDK1 only inhibited AKT Thr308 phosphorylation but not Ser473 phosphorylation (Fig. S4A). Moreover, knockdown of SYK impaired glucose starvation-induced PDK1 dimerization, while knockdown of PDK1 did not affect SYK phosphorylation (Fig. S4B, C). Collectively, considering that PI3K-mediated PIP3 generation is also necessary for PDK1 activation [41], we conclude that SYK-mediated AKT activation may represent the predominant mechanism.

Finally, to investigate the generality of the mechanism underlying AKT activation, we examined the phosphorylation of SYK and the dimerization of PDK1 in response to glutamine starvation, CCCP treatment, and CQ treatment. Our results demonstrated that both the phosphorylation of SYK and the dimerization of PDK1 were significantly enhanced under conditions of glutamine starvation, whereas they were only mildly affected by CCCP or CQ treatment (Fig. S4D, E). Given that tumor cells heavily depend on glucose and glutamine metabolism, it can be reasonably concluded that SYK activation and PDK1 dimerization-mediated AKT activation occur commonly under conditions of both glucose and glutamine deprivation.

## Discussion

**Fig. 7:**
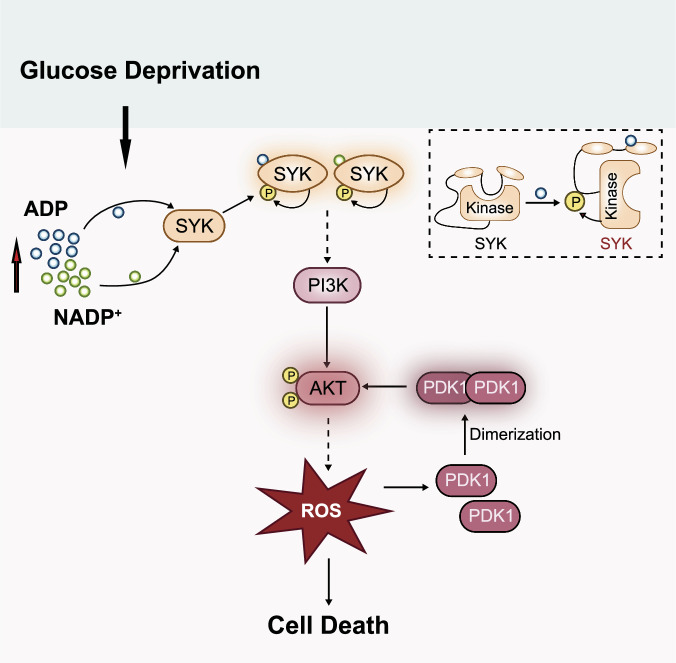
A model for the activation of AKT under conditions of glucose starvation through a dual mechanism. Glucose deprivation leads to increased levels of ADP and NADP^+^, which directly bind to SYK, inducing a conformational change that results in the self-activation of SYK. The activated SYK subsequently initiates PI3K-dependent activation of AKT. Additionally, the rise in ROS levels due to glucose deprivation promotes the oxidative dimerization of PDK1, enhancing its ability to recognize and activate AKT. In melanoma cells, AKT is crucial for increasing ROS levels and inducing cell death during glucose deprivation.

The proto-oncogene AKT, which is activated in multiple cancers, functions as an anti-apoptotic factor in response to various stimuli such as radiation, hypoxia, and chemotherapy [42, 43]. However, its roles under metabolic stress remain elusive. In this study, we demonstrated that AKT was significantly activated under conditions of glucose deprivation in various cancer cell lines. Mechanistically, glucose deprivation increased the levels of ADP and NADP^+^, which could be sensed by SYK through direct interaction. This binding of ADP or NADP^+^ promoted a conformational alteration of SYK, leading to self-phosphorylation and activation of SYK. The activated SYK enhanced the activity of PI3K, resulting in PI3K-dependent activation of AKT. In melanoma cells, the SYK-AKT axis played a critical role in ROS elevation and cell death during glucose deprivation. Moreover, the elevated ROS facilitated dimerization and activation of PDK1, thereby further activating AKT through a feedforward mechanism (Fig. 7). Overall, our study not only establishes a significant correlation between energy insufficiency and activation of the SYK-AKT axis but also provides insights into the role of AKT in sensitizing cells to metabolic stress.

AKT is activated by various growth factors, such as IGF and EGF, which bind to their corresponding receptor tyrosine kinases (RTKs) to stimulate the activation of PI3K. Subsequently, PI3K catalyzes the production of phosphatidylinositol-3,4,5-triphosphate (PIP3), serving as a second messenger that facilitates AKT activation [44]. Although PI3K activity and PIP3 elevation are essential for AKT activation during glucose deprivation, RTKs do not seem to participate in this process. Instead, SYK links glucose deprivation to PI3K activation. SYK has been identified as an upstream activator of PI3K [10, 18]. Typically, SYK is activated by classical immunoreceptors, including B cell receptors (BCRs), T cell receptors (TCRs), and Fcγ receptors. Phosphorylation of the immunoreceptor tyrosine-based activation motif (ITAM) within these receptors creates docking sites for the SH2 domain of SYK. This interaction induces a conformational change in SYK leading to its autophosphorylation and subsequent activation [45]. The activation of SYK through phosphorylated ITAM-mediated pathways highlights the importance of the binding between the SH2 domain of SYK and phosphorylated groups. Our findings demonstrate a direct interaction between the SH2 domain of SYK and ADP or NADP^+^, two metabolites containing diphosphate groups, which may mimic the interaction with phosphorylated ITAM, leading to conformational changes and self-activation of SYK in an ITAM independent manner. Despite the substantial rise in ADP and NADP^+^ concentrations during glucose deprivation, the NADP^+^ level (~15 μM) remains significantly below its Kd for SYK (~69 μM). Conversely, the ADP concentration (~110 μM) is markedly above its Kd value for SYK (~42 μM). Consequently, it is plausible that SYK predominantly responds to intracellular ADP levels, thereby linking nutrient scarcity to the activation of the PI3K-AKT pathway.

The PI3K-AKT pathway is a critical signaling cascade that plays a significant role in cell survival and proliferation by inhibiting apoptosis, enhancing protein translation, and regulating glucose metabolism [4, 46]. However, it has been suggested that the ability of AKT to inhibit cell death may depend on glucose metabolism [47, 48]. For instance, AKT only suppresses Bim-induced cell death when glucose is present [49]. An increasing number of studies have shown that under metabolic stress conditions, AKT activation does not inhibit cell death but rather accelerates it [50]. Under conditions of glucose deprivation, AKT activation promotes the generation of ROS. Given the substantial decrease in the levels of the reducing equivalent NADPH, which is essential for antioxidant defense, during glucose deprivation [38], this AKT-facilitated ROS production may bring cancer cells closer to the threshold for ROS-induced cell death. Although the exact mechanism by which AKT facilitates the elevation of ROS remains to be determined in future research, it is plausible that AKT-mediated activation of mTOR or inhibition of FOXO3 may be involved [50]. Previous studies have reported that mTOR activation enhances oxygen consumption and increases ROS levels [51], while FOXO3 activation is associated with the transcriptional regulation of antioxidant-related genes [52, 53]. Additionally, since we specifically tested melanoma cells expressing predominantly the AKT3 isoform in our study, we cannot exclude the possibility that it is specifically AKT3 rather than other isoforms promoting cell death under glucose deprivation conditions due to distinct biological functions reported for different members within the AKT family [5, 54].

Interestingly, this AKT-facilitated elevation of ROS also triggers the activation of AKT through a feedforward mechanism. In this mechanism, ROS stimulates PDK1 to further enhance the phosphorylation of AKT at Thr308. It is known that PDK1 is activated upon binding to PIP3, which is generated by PI3K [41]. In our study, we demonstrate that ROS-mediated dimerization of PDK1 in a Cys21 and Cys23 dependent manner may serve as an additional mechanism for PDK1 activation and subsequent AKT phosphorylation. Consistent with previous reports indicating that dimerization of PDK1 facilitates its recognition of substrate protein kinase C (PKC) [55], the Cys21-Cys23 mediated dimerization promotes the interaction between PDK1 and AKT, thereby leading to AKT phosphorylation. Further investigation is warranted to determine whether this oxidative dimerization also plays a role in recognizing other substrates of PDK1.

In conclusion, upon glucose deprivation, AKT is activated through a dual mechanism involving ADP/NADP^+^-mediated activation of the SYK-PI3K axis and ROS-mediated activation of PDK1. As such, AKT not only serves as a kinase triggered by growth factors but also potentially operates as an indirect sensor of energy status during metabolic stress.

## Materials and methods

### Cell culture

The human embryonic kidney (HEK293T) cells; human melanoma cell lines MV3, Mel-RM, A375; hepatoma cell lines Hepa1-6, HepG2; colorectal carcinoma cell lines HCT116, SW620 and human breast cancer cell lines MDA-MB-231, PY8116 were cultured in Dulbecco’s Modified Eagle Medium (DMEM)-high glucose (Sigma) supplemented with 10% fetal bovine serum (FBS, Lonsera BIOPRODUCTS), 100 IU penicillin and 100 mg/mL streptomycin (Bio Basic Inc). Mouse mammary carcinoma cells 4T1 were maintained in RPMI-1640 (Thermo Fisher Scientific) supplemented with 10% FBS, 100 IU penicillin, and 100 mg/ml streptomycin. The concentration of FBS remains at 10% during various drug treatments.

### Glucose deprivation

One day prior, cells of an appropriate density were plated in a 12-well plate. On the following day, the DMEM-high glucose (Sigma) culture medium was removed, and the cells were rinsed once with DMEM-without glucose culture medium (Sigma). Subsequently, glucose-deficient culture medium was added to the wells of the experimental group for 3 h to assess AKT phosphorylation and PDK1 dimerization levels, 1 h to evaluate SYK phosphorylation levels, 6 h to detect ROS levels, and 9 h to assess cell viability. If pharmacological stimulation is needed, it was administered concurrently with glucose deprivation.

### Plasmid construction

AKT1, AKT2, AKT3, SYK, PDK1, and their mutants were cloned and inserted into p3xFlag-CMV10, pCMV5-HA, pCDH-EF1-MCS, or pGEX4T1 vectors using human embryonic kidney (HEK293T) cells cDNA as templates. The pT7-6xHis-BCAP was purchased from Miaoling Biology, China. The siRNA-resistant or sgRNA-resistant mutants were generated by introducing a silent mutation at the siRNA or sgRNA target sites. The PDK1^9CS^, PDK1^C21/23S^, PDK1^S21/23A^, and SYK^5A^ (R22A, R42A, R45A, D46A, K247A) mutants were generated through LIC using primers carrying corresponding target site mutations and subsequently verified by sequencing.

### Generation of the lentiviral system

The lentiviral-based vector plko.1 was used to express shRNAs in melanoma cells, and lentiCRISPR v2 was used to express sgRNAs in melanoma cells that expressed cas9. Lentiviruses were generated by transfecting HEK293T cells with the lentiviral backbone constructs, the packaging plasmid psPAX2 (Addgene 12260) and the envelope plasmid pMD2.G (Addgene 14887) (4 μg:3 μg:1 μg for 6-cm dishes) using the calcium phosphate transfection method. After transfection for 8–12 h, the medium was removed, and fresh warm medium containing non-essential amino acids was added. Viral supernatants were collected 48 h after the transfection. Freshly plated melanoma cells were infected with the lentivirus, and the knockdown or knockout efficiency for the target genes was determined by western blotting or RT-PCR. For detection of knock out efficiency by qPCR, the primers were designed to span Cas9 nuclease cutting site near its 3′ end to render qPCR the ability in selectively amplifying wild type sequence. The sequences of sgRNAs or shRNA are as follows(5′-3′):

shSYK-1: TTGGTCAGCGGGTGGAATAAT,

shSYK-2: TACCCAACATTACGCCAAGAT,

sgPDK1-1: CATTTATGTTTCTGCGGCAA,

sgAKT1-1: TGTCATGGAGTACGCCAACG,

sgAKT1-2: TCACGTTGGTCCACATCCTG,

sgAKT2-1: GACCCCATGGACTACAAGTG,

sgAKT2-2: CTCTTGAGTACTTGCACTCG,

sgAKT3-1: CTGCACCATAGAAACGTGTG,

sgAKT3-2: ATTTCATGTAGATACTCCAG,

shPIK3CA-1: GCAATTTGGGTAGAATTTCG,

shPIK3CA-2: GTTCGAACAGGTATCTACCA,

shPIK3CD: AGGAGCCTACGTGGCAATCG.

### RT-PCR and primers

Total RNA was extracted using a total RNA isolation kit (Vazyme, China) and reverse transcribed using a reverse transcriptase kit (ABclonal, China). cDNA was used as a template for the amplification, and the level of β-actin was used as a normalization control. The primer sequences used were as follows (5′-3′):

AKT1-Forward: TGTCATGGAGTACGCCAACG,

AKT1-Reward: TCCCTCCAAGCTATCGTCCA,

AKT2-Forward: GACCCCATGGACTACAAGTG,

AKT2-Reward: CCTGGTTGTAGAAGGGCAGG,

AKT3-Forward: CACACGTTTCTATGGTGCAG,

AKT3-Reward: TTCATGGTGGCTGCATCTGT.

### Reagents and antibodies

Deoxy-D-Glucose (2-DG, A602241) and Glutathione Reduced (GSH, A100399) were purchased from Sangon biotech. CCCP (C2759), BAPTA-AM (A1076), N-Acetyl-L-cysteine (NAC, A7250) and Propidium Iodide(PI, P4170) were purchased from Sigma Aldrich. 3× Flag peptide (HY-P0319A), BAY876 (HY-100017), Chloroquine (CQ, HY-17589A), Vo-ohpic (HY-110067), PRT062607 (HY-15322), R406 (HY-12067), H89 (HY-15979), Gallein (HY-D0254), NT157 (HY-100037), Pyridone (HY-14435), Lonafarnib (HY-15136), SB505124 (HY-13521) and Src-inhibitor I (HY-101053) were purchased from MedChem Express. Glutamine (G105425), ADP (A119474), NADP^+^ (N107170), NADPH (V276146), and AMP-Na2 (A100357) were purchased from Aladdin. NAD^+^ (T1609) was purchased from Target Mol. Streptolysin O (SLO) (S4470) was purchased from Solarbio. CM-H_2_DCFDA (C6827) and Cell Rox Green (C10444) were purchased from Thermo Fisher Scientific.

Goat anti-rabbit (31210), at a 1:5,000 dilution for the immunoblot (IB), and anti-mouse (31160, 1:5,000 dilution for IB) secondary antibodies were purchased from Thermo Fisher Scientific. Anti-tubulin (T-4026, 1:5,000 dilution for IB), anti-HA (mouse) (H-9658, 1:5000 dilution for IB), and anti-Flag (mouse) (F-1804, 1:5000 dilution for IB and 1:200 dilution for immunofluorescence (IF)) antibodies were purchased from Sigma Aldrich. Anti-AKT (40D4, 1:2000 dilution for IB), anti-Phos-AKT(Ser473) (D9E, 1:2000 dilution for IB), anti-Phos-AKT(Thr308) (D25E6, 1:2000 dilution for IB), anti-SYK (Rabbit) (D3Z1E, 1:2000 dilution for IB), anti-SYK (Mouse) (4D10, 1:2000 dilution for IB), anti-PDK1 (D4Q4D, 1:2000 dilution for IB) and anti-Phospho-Tyr (P-Tyr-100, 1:1000 dilution for IB) antibodies were purchased from Cell Signaling Technology. Anti-Phos-SYK-Tyr348 (YP0614,1:1000 dilution for IB) antibody was purchased from Immunoway. Anti-LDHA (A0861, 1:2000 dilution for IB) antibody was purchased from Abclonal. Anti-TFR1 (10084-2-AP, 1:2000 dilution for IB) and anti-His (1B7G5) antibodies were purchased from Proteintech. Anti-GST (M20007, 1:5000 dilution for IB) antibody was purchased from Abmart.

### Immunoprecipitation and western blot analysis

Cells were lysed in lysis buffer (20 mM Tris; 150 mM NaCl; 1 mM Na_2_EDTA; 1 mM EGTA; 2.5 mM Sodium pyrophosphate; Triton-100 1% and 1 mM PMSF). Proteins were immunoprecipitated from the cell lysates by incubating the lysates with the appropriate antibodies and protein A/G agarose beads for 3 h at 4 °C. The immunoprecipitants were then collected and washed three times with lysis buffer to perform western blot analysis. The samples were separated by SDS-PAGE, transferred to a PVDF membrane, and then analyzed by immunoblotting with the specific antibodies.

### PIP3 measurements

The amounts of PIP3 in cells were measured by human PIP3 ELISA kit (MEIMIAN, China). Ultrasonicated cells were concentrated to a minimum of 1 million cells per ml, followed by centrifugation at 3000 × *g* for 20 min at 4 °C to collect the supernatant. The collected supernatant was then incubated with ELISA coated plates. The OD value at 450 nm was measured using a Microplate Reader (TECAN).

### Cells viability analysis

Cells survival rates were analyzed by propidium iodide (PI) staining. Briefly, cells were harvested and washed in PBS. The cells were then resuspended in 1 ml PBS containing 5 μg/μl propidium iodide. Propidium iodide incorporation was quantified using an FC500 (Beckman) flow cytometer. Propidium iodide-negative cells that were of normal size were considered viable cells.

### ROS level measurement

The ROS levels were measured using the Cell-Rox Green Reagent. The cells were cultured in DMEM or glucose-free mediums for 6 h, followed by incubating with Cell-Rox Green Reagent protect from light at a final concentration of 2.5 μM for 30 min at 37 °C in 5% CO_2_. Collected and resuspended cells in ice-cold PBS for ROS analysis using an FC500 (Beckman) flow cytometer.

### Cells membrane components isolation

Cells in 6 cm plates were harvested using 300 μl buffer (75 mM NaCl; 1 mM NaH_2_PO4; 8 mM Na_2_HPO4; 250 mM saccharose) with 1 mM PMSF to 1.5 ml tube and placed on ice, and a sample of 60 μl was taken as the input group. The liquid residual was mixed with an additional 300 μl of buffer containing digitonin (380 μg/ml) and kept on ice for 5 min. The mixture was centrifuged at 4 °C and at a speed of 12,000 × *g* for 5 min. The precipitation containing membrane components was resuspended in 400 μl of buffer and washed for twice. The precipitate was subjected to ultrasonication in 120 μl lysis buffer (20 mM Tris; 150 mM NaCl, 1 mM Na_2_EDTA, 1 mM EGTA, 2.5 mM Sodium pyrophosphate, Triton-100 1%) before Western blot analysis.

### MicroScale thermophoresis (MST) analysis

Experiments were performed using Monolith NT.115 (Blue/Red) instrument (NanoTemper Technologies GmbH, Germany). The cell lysates of HEK293T cells expressing SYK-EmerGFP, N-Terminal-EmerGFP, or SYK^5A^-EmerGFP, respectively, were used as source of fluorescently labeled targets. HEK293T cells were transfected with GFP-fused protein as described and lysed 24 h after transfection. To evaluate binding of ADP or NADP^+^ to target protein, cell lysates were diluted 1.5 times with lysis buffer (20 mM Tris; 150 mM NaCl; 1 mM Na_2_EDTA; 1 mM EGTA; 2.5 mM Sodium pyrophosphate; Triton-100 1%) to provide optimal level of fluorescence. ADP or NADP^+^ as ligands were incubated with diluted cell lysates. The measurements were conduced in capillaries (NanoTemper Technologies GmbH, MO-K025) using LED source with 470 nm and <30% infrared-laser power at 25 °C. Each data point represents mean ΔFnorm values from three independent experiments. The bound fractions of the SYK-EmerGFP, N-Terminal-EmerGFP, or SYK^5A^-EmerGFP were determined, and the fitting was performed using the Hill model method incorporated by the MO Affinity Analysis v2.3 software (NanoTemper Technologies).

### Cell permeabilization assay

The cells were pretreated with SLO (Streptolysin O, final concentration 50 ng/ml) in permeabilization buffer (137 mM NaCl; 3 mM KC; 2 mM MgCl_2_; 5.6 mM glucose; 0.1 mg/ml BSA; l00 nM Ca^2+^; 3 mM EGTA) for 10 mins, followed by incubating cells with AMP (200 μM), ADP (200 μM), NAD^+^ (200 μM) or NADP^+^ (100 μM) for a certain amount of time in permeabilization buffer without SLO.

### Metabolite analysis by LC-MS

A375 cells (1.5 × 10^6^) were treated with DMEM or glucose-free medium for 1 h. After washing for three times with PBS, 1 ml of extraction solution (methanol: acetonitrile: water = 2:2:1) was added to cells, followed by vortex agitation for 30 s and ultrasonication in ice for 10 min. Freeze-thaw cycles were performed in liquid nitrogen, followed by another round of ultrasound in ice for 10 min; this process was repeated three times. The mixture was then incubated at −20 °C for 1 h to fully remove proteins. After centrifugation at 13,000 rpm and 4 °C for 15 min, the supernatant was concentrated and spin-dried using a low-temperature vacuum centrifugal concentrator. Finally, both the samples and a set of five gradient dilution standard samples containing AMP, ADP, NAD^+^, and NADP^+^ components were subjected to LCMs (AB SCIEX QTRAP6500+) analysis to determine the absolute quantities of these metabolites in A375 cells. The average cell volume(Control group: 4357 μm^3^, -Glc group (1 h): 3709 μm^3^, -Glc group(2 h):1480 μm^3^) was determined by Imaris X64 9.2.0 software (Bitplane) from the axial image stacks of CM-H_2_DCFDA(Thermo Fisher Scientific, 2600176) labeled A375 cells taken under Evidence IXplore SpinSR.

### Protein conformation alteration assay

SspB-Flag plasmid and SYK-N11-SsrA plasmid were constructed as previously reported [35]. A375 cells expressing SspB-Flag and SYK-N11-SsrA were glucose starved or treated with ADP or NADP^+^, followed by harvesting cells in lysis buffer (20 mM Tris; 150 mM NaCl; 1 mM Na_2_EDTA; 1 mM EGTA; 2.5 mM Sodium pyrophosphate; Triton-100 1%; 1 mM PMSF). SspB-Flag proteins were immunoprecipitated from the cell lysates by incubating the lysates with anti-Flag antibody and protein A/G agarose beads. The level of SYK-N11-SsrA in immunoprecipitants was determined by Western blot analysis.

### In vitro kinase assay

The SYK-Flag protein immunoprecipitated from control or glucose starved A375 cells or bacterial expression SYK was incubated with bacterial expressed BCAP-6×His in kinase reaction buffer (10 mM Tris-HCl (pH 7.5); 10 mM MgCl_2_; 10 mM MnCl_2_; 1 mM EGTA; 2 mM DTT; 100 μM ATP) for 60 min at 30 °C. The reaction was stopped by loading buffer and boiling the mixture for 10 min. Substrate phosphorylation was subjected to western blot analysis using anti-phospho-Tyr antibody.

For PDK1 kinase assay, PDK1-HA was immunoprecipitated from control or glucose starved A375 cells, and then incubated with bacterial expressed AKT-GST in kinase reaction buffer (10 mM Tris-HCl (pH 7.5), 10 mM MgCl_2_, 10 mM MnCl_2_, 1 mM EGTA, 100 μM ATP) for 30 min at 37 °C. The reaction was stopped by loading buffer and boiling the mixture for 10 min. Substrate phosphorylation was subjected to western blot analysis using anti-AKT-Thr308 antibody.

### Statistical analysis

The data are expressed as the mean ± SEM, and the statistical analysis of differences between two groups was performed using two-tailed Student’s t-test. The differences between multiple groups were analyzed using one-way or two-way ANOVA, followed by Tukey’s multiple comparison test. The statistical analysis was performed using GraphPad Prism 6. *P* < 0.05 was considered statistically significant.

## Supplementary information

Supplementary figures

Uncropped Western blot images

qPCR source data

## Data Availability

All data generated or analyzed during this study are included in this published article (and its supplementary information files).
