# Supplementary material for: Activation of AKT via a dual mechanism enhances the susceptibility of melanoma cells to glucose deprivation
Source: Cell Death Dis. 2025 Aug 7;16(1):595. doi: 10.1038/s41419-025-07906-4 (PMC12331947; doi:10.1038/s41419-025-07906-4)
Supplement: Supplementary file 1 — Supplementary figures [file 41419_2025_7906_MOESM1_ESM.pdf]

Figure S1

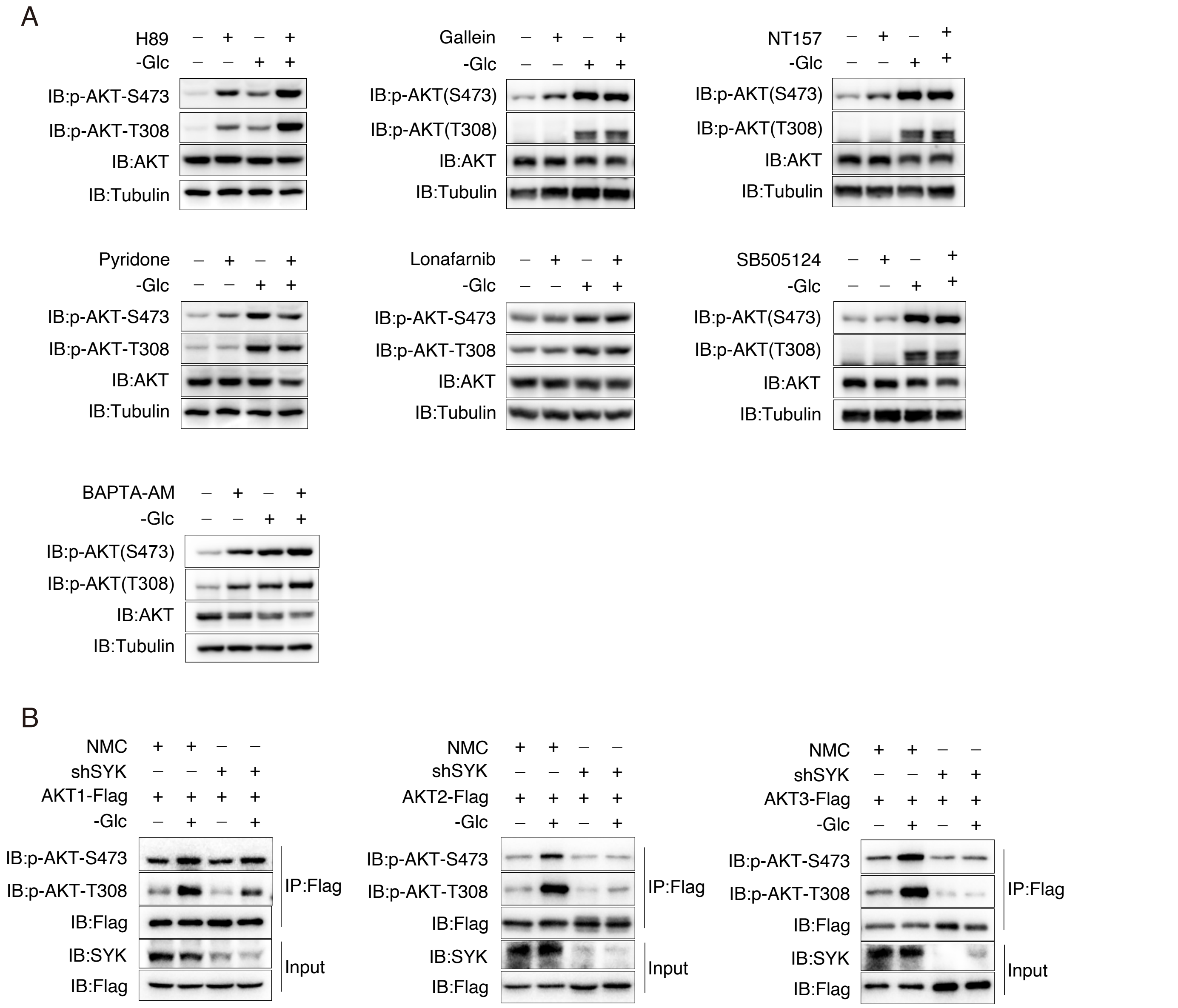

A. Screening of inhibitors for glucose starvation-induced AKT phosphorylation. A375 cells were treated with or without different inhibitors including H89 (10 μM), Gallein (10 μM), NT157 (5 μM), Pyridone (5 μM), Lonafarnib (5 μM), SB505124 (5 μM) and BAPTA-AM (5μM) while being cultured under glucose starvation for 3 hours, then the phosphorylation of AKT was determined.

B. SYK is critical for the activation of AKT family members upon glucose starvation. Flag-tagged AKT1, AKT2, and AKT3 were transfected into control or SYK-knockdown A375 cells. The phosphorylation levels of AKTs under glucose starvation for 3 hours were detected following immunoprecipitation of Flag-AKTs.

Figure S2

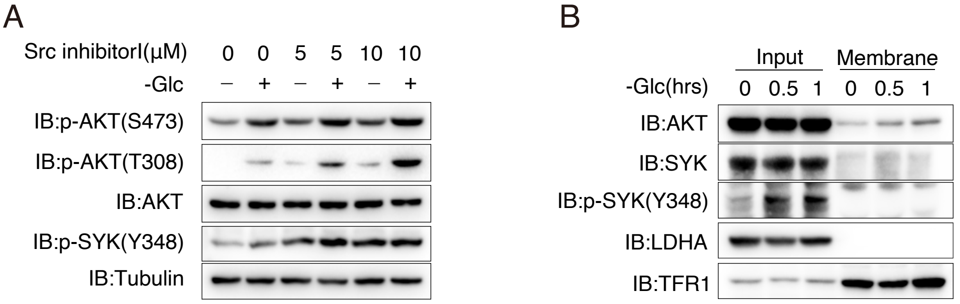

A. Src inhibitor cannot block glucose starvation-induced AKT activation. The A375 cells were treated with Src inhibitor I at the indicated concentration during glucose starvation for 3 hours. The phosphorylation of AKT and SYK was assessed.

B. Glucose deprivation does not affect the level of SYK on cell membrane. Mel-RM melanoma cells were cultured in a glucose-free medium for the indicated durations. The protein levels of AKT, SYK, and phosphorylated SYK in the membrane fraction were determined. Lactate dehydrogenase A (LDHA) and transferrin receptor 1 (TFR1) served as markers for cytoplasm and membrane, respectively.

Figure S3

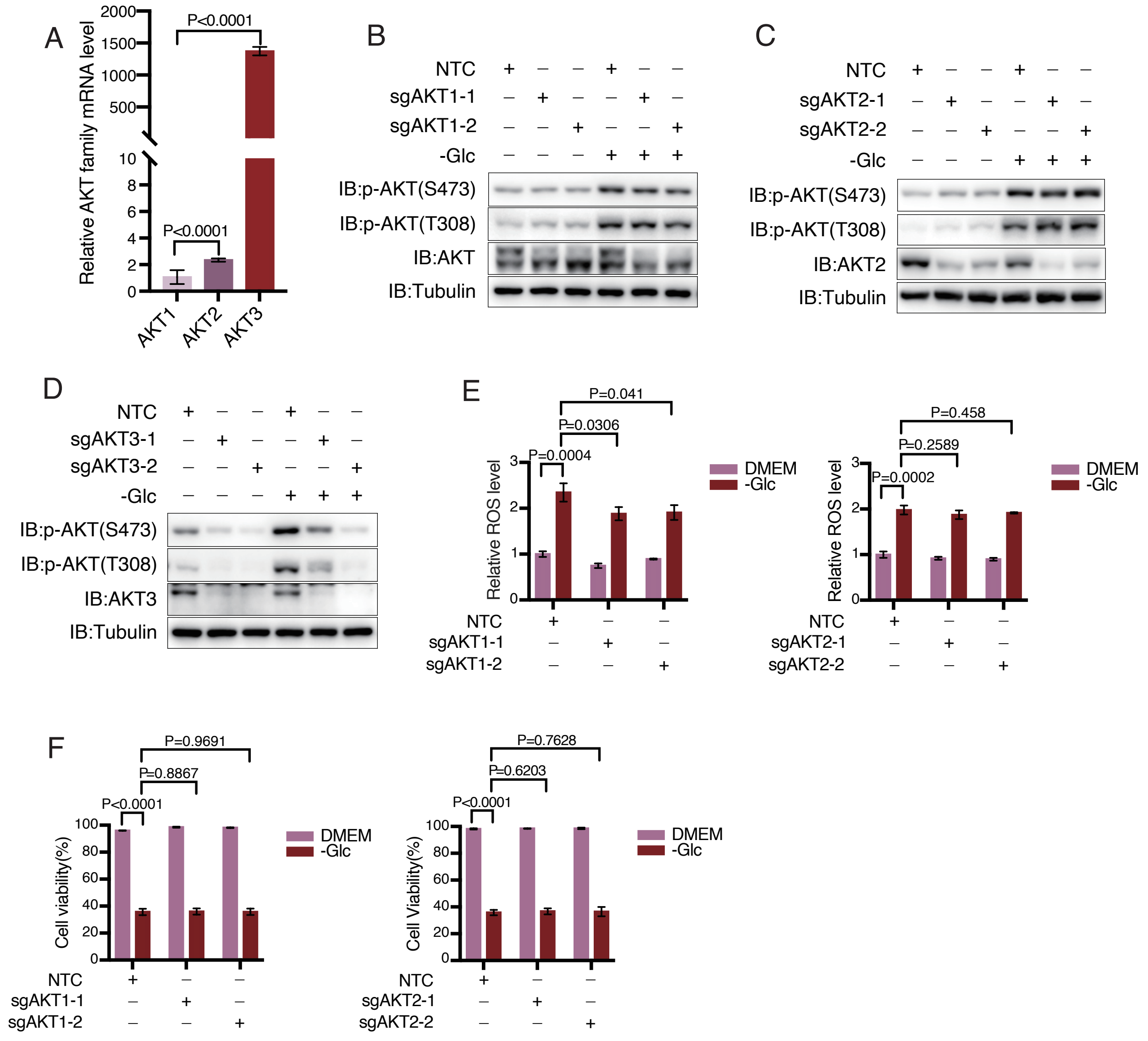

A. The expression of AKT3 is higher compared to AKT1 and AKT2 in A375 cells. The mRNA level of the AKT family members were determined by qPCR in A375 cells.

B-D. Knockout of AKT3 impaired glucose starvation-induced AKT phosphorylation level. The CRISPR/Cas9 approach was employed to knock out AKT1 (B), AKT2 (C), or AKT3 (D) in A375 cells. The phosphorylation of pan-AKT was assessed following a 3hour period of glucose deprivation.

E, F. Knock out of AKT1 or AKT2 does not affect glucose starvation-induced ROS elevation and cell death. The AKT1-KO or AKT2-KO A375 cells were cultured in a glucose-free medium for 6 hours to assess ROS level (E), and for 9 hours to analyze cell viability (F).The data are presented as the means ± SEM of three independent experiments

Figure S4

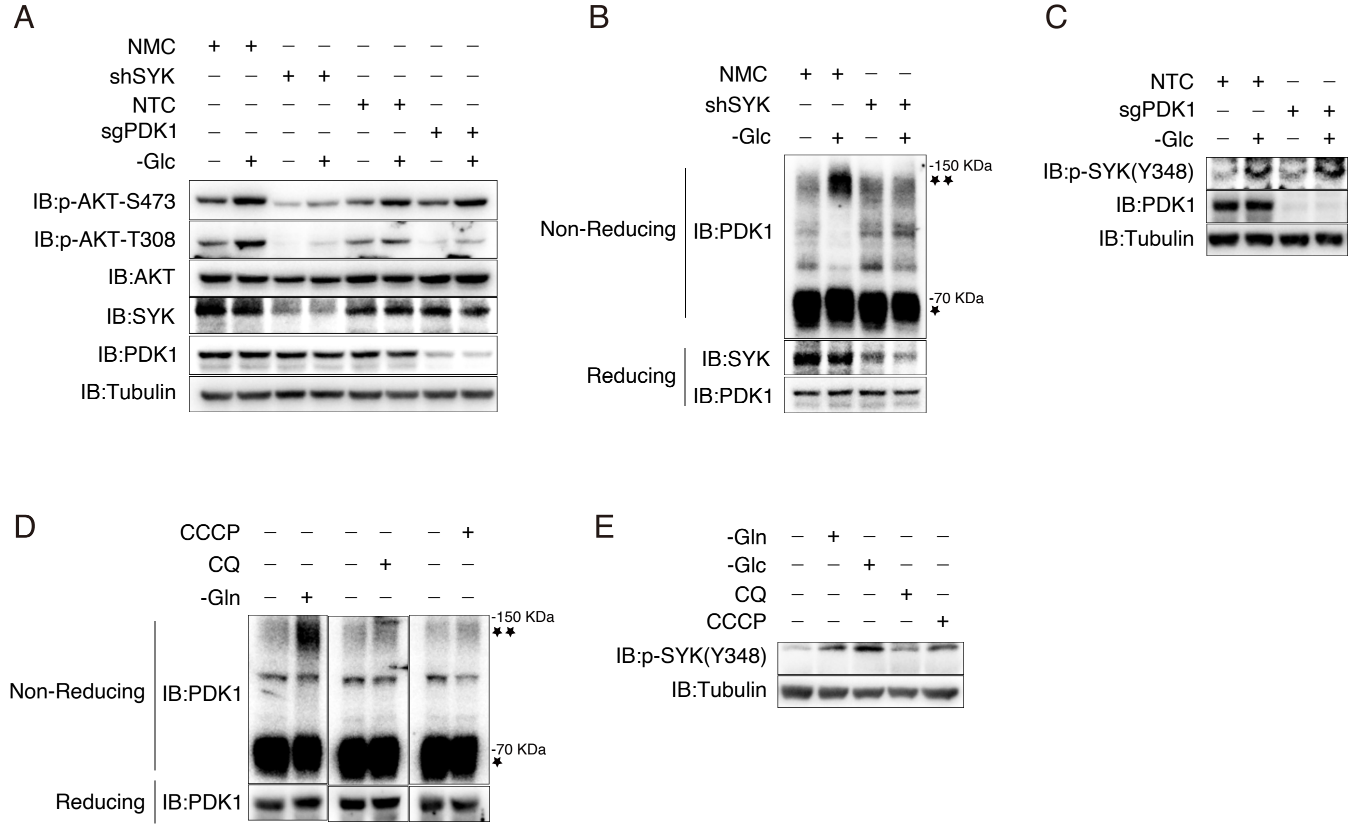

A. Both SYK and PDK1 are important for AKT activation upon glucose starvation. The A375 cells with SYK knockdown or PDK1 knockout were cultured under glucose starvation conditions for 3 hours to measure the phosphorylation of AKT.

B. SYK is important for PDK1 dimerization upon glucose starvation. Control or SYK knockdown A375 cells were glucose starved for 3 hours. The oxidative dimerization of PDK1 were detected under non-reducing conditions.

C. PDK1 is indispensable for glucose starvation-induced SYK phosphorylation. Control or PDK1 knockout A375 cells were glucose starved for 3 hours. The phosphorylation of SKY at Tyr348 were determined.

D. PDK1 is dimerized under energy deficiency. A375 cells were cultured under glutamine deprived medium, or treated with CCCP (2.5 µM) or CQ (20 µM) for 3 hours. The oxidative dimerization of PDK1 were detected under non-reducing conditions.

E. SYK is phosphorylated under energy deficiency. A375 cells were cultured under glutamine deprived medium, or treated with CCCP (2.5 µM) or CQ (20 µM) for 3 hours. The phosphorylation of SKY at Tyr348 were then determined.
