# Supplementary material for: Activation of AKT via a dual mechanism enhances the susceptibility of melanoma cells to glucose deprivation
Source: Cell Death Dis. 2025 Aug 7;16(1):595. doi: 10.1038/s41419-025-07906-4 (PMC12331947; doi:10.1038/s41419-025-07906-4)

Uncropped western blot images
Figure1

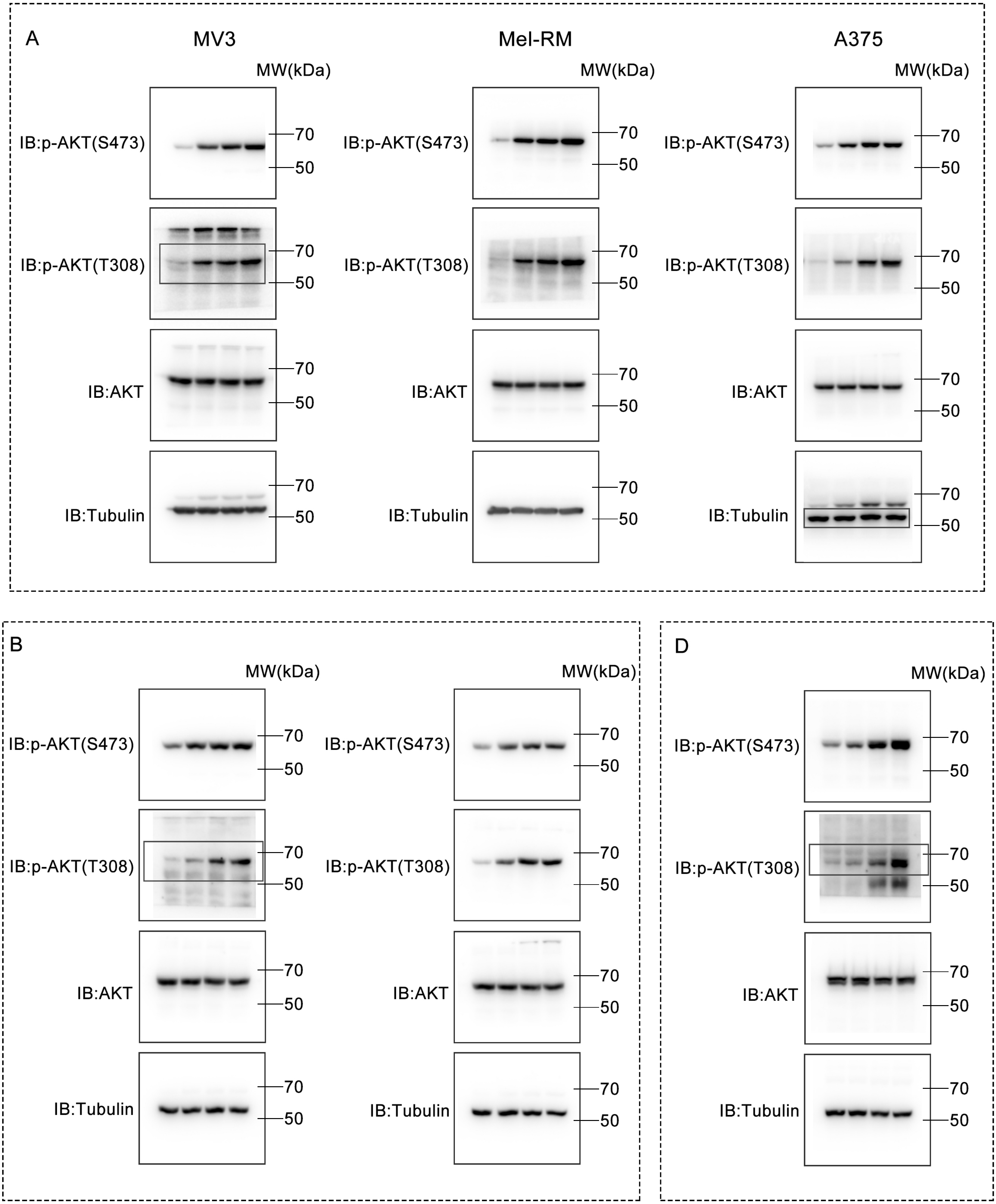

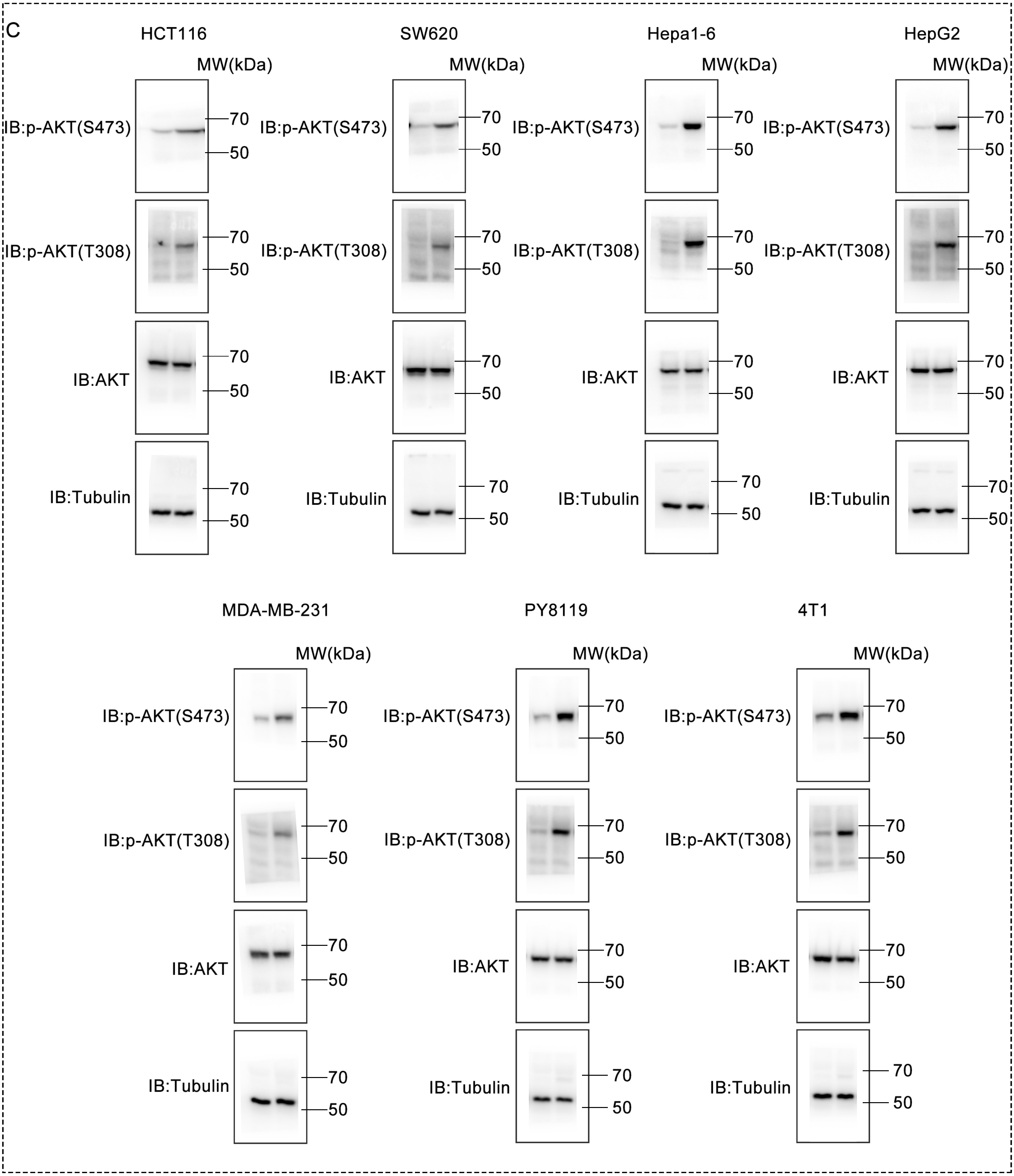
C
HCT116
SW620
Hepa1-6
HepG2
MW(kDa)
IB:p-AKT(S473)
IB:p-AKT(T308)
IB:AKT
IB:Tubulin
70
50
MDA-MB-231
PY8119
4T1

E

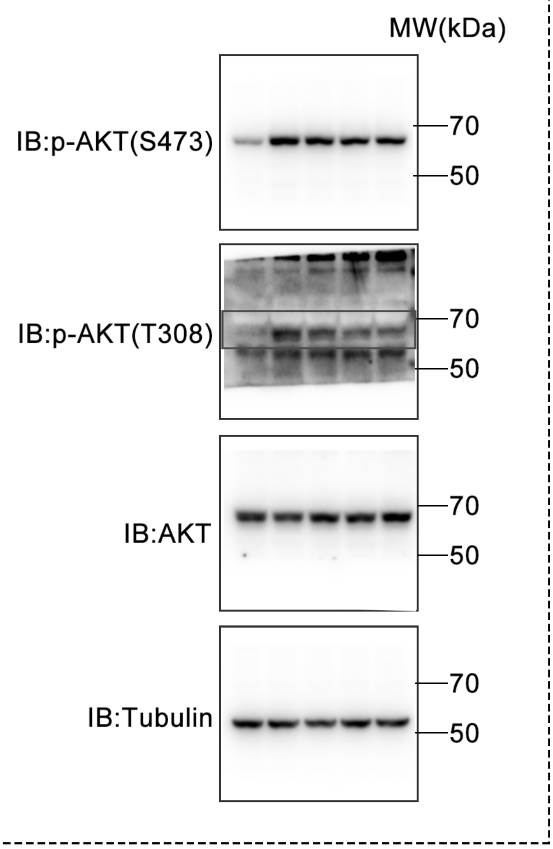
MW(kDa)
IB:p-AKT(S473)
70
50
IB:p-AKT(T308)
70
50
IB:AKT
70
50
IB:Tubulin
70
50

F

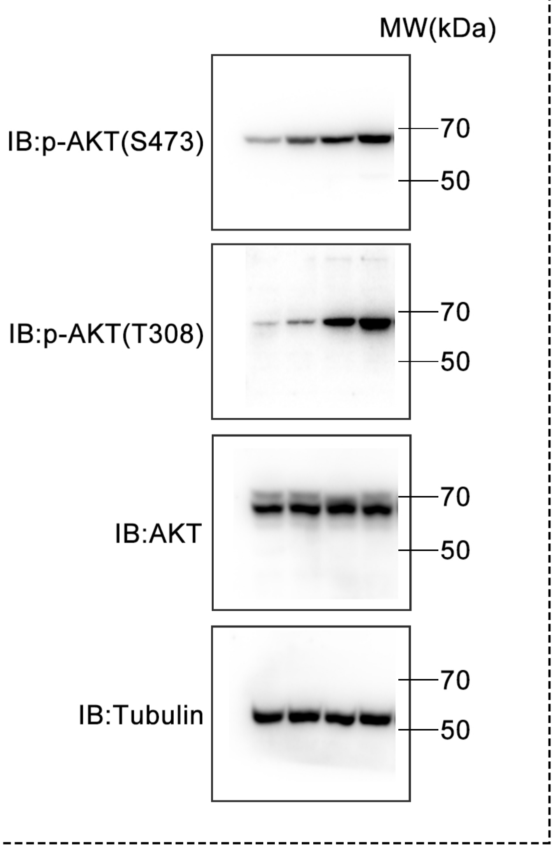
MW(kDa)
IB:p-AKT(S473)
70
50
IB:p-AKT(T308)
70
50
IB:AKT
70
50
IB:Tubulin
70
50

G

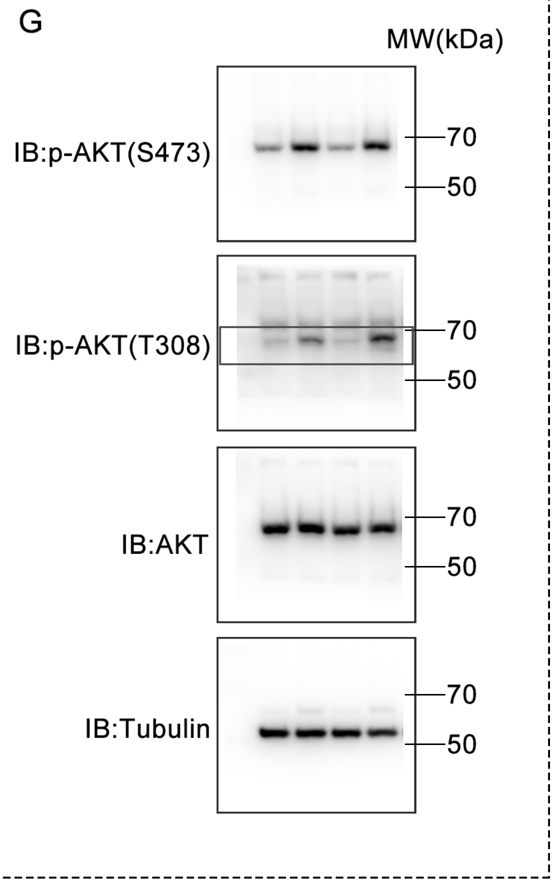
MW(kDa)
IB:p-AKT(S473)
70
50
IB:p-AKT(T308)
70
50
IB:AKT
70
50
IB:Tubulin
70
50

H

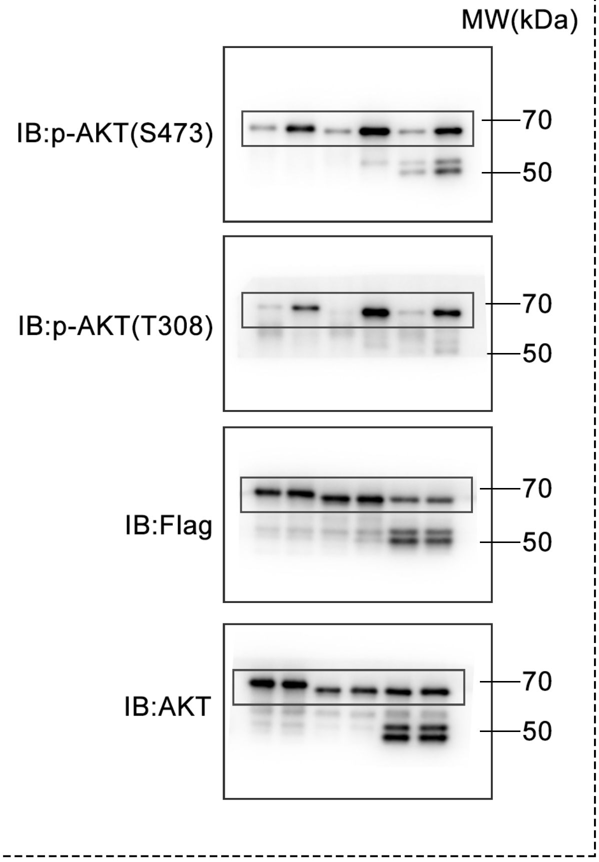
MW(kDa)
IB:p-AKT(S473)
70
50
IB:p-AKT(T308)
70
50
IB:Flag
70
50
IB:AKT
70
50

Uncropped western blot images
Figure 2

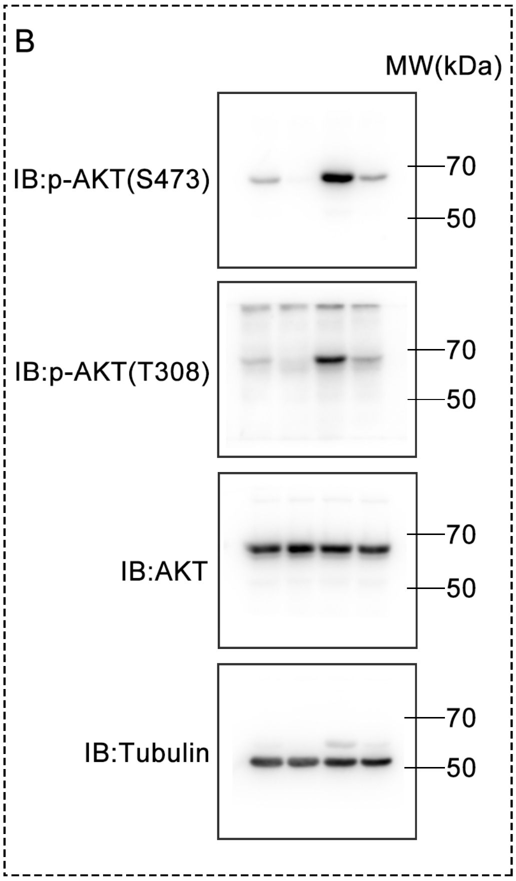

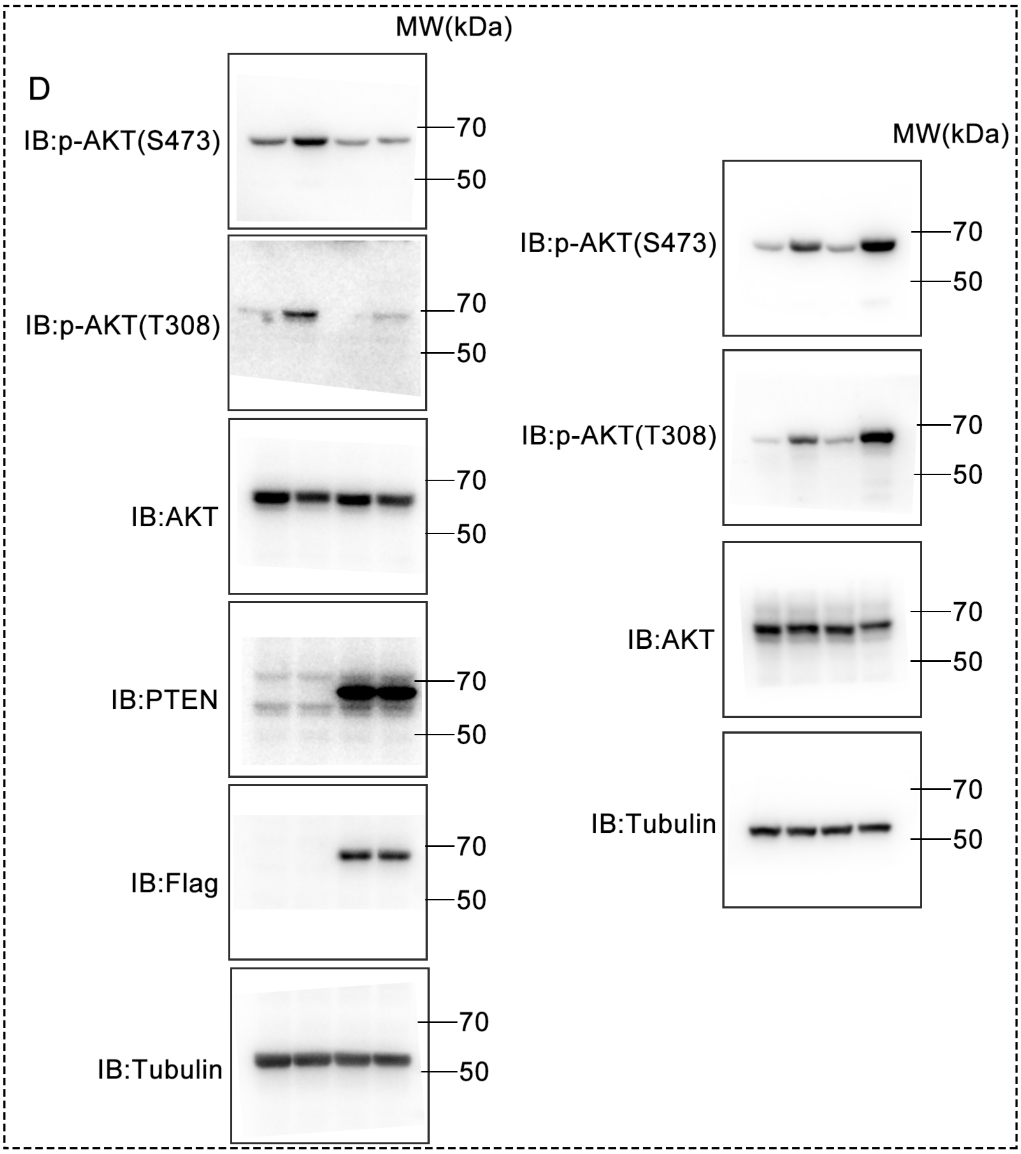

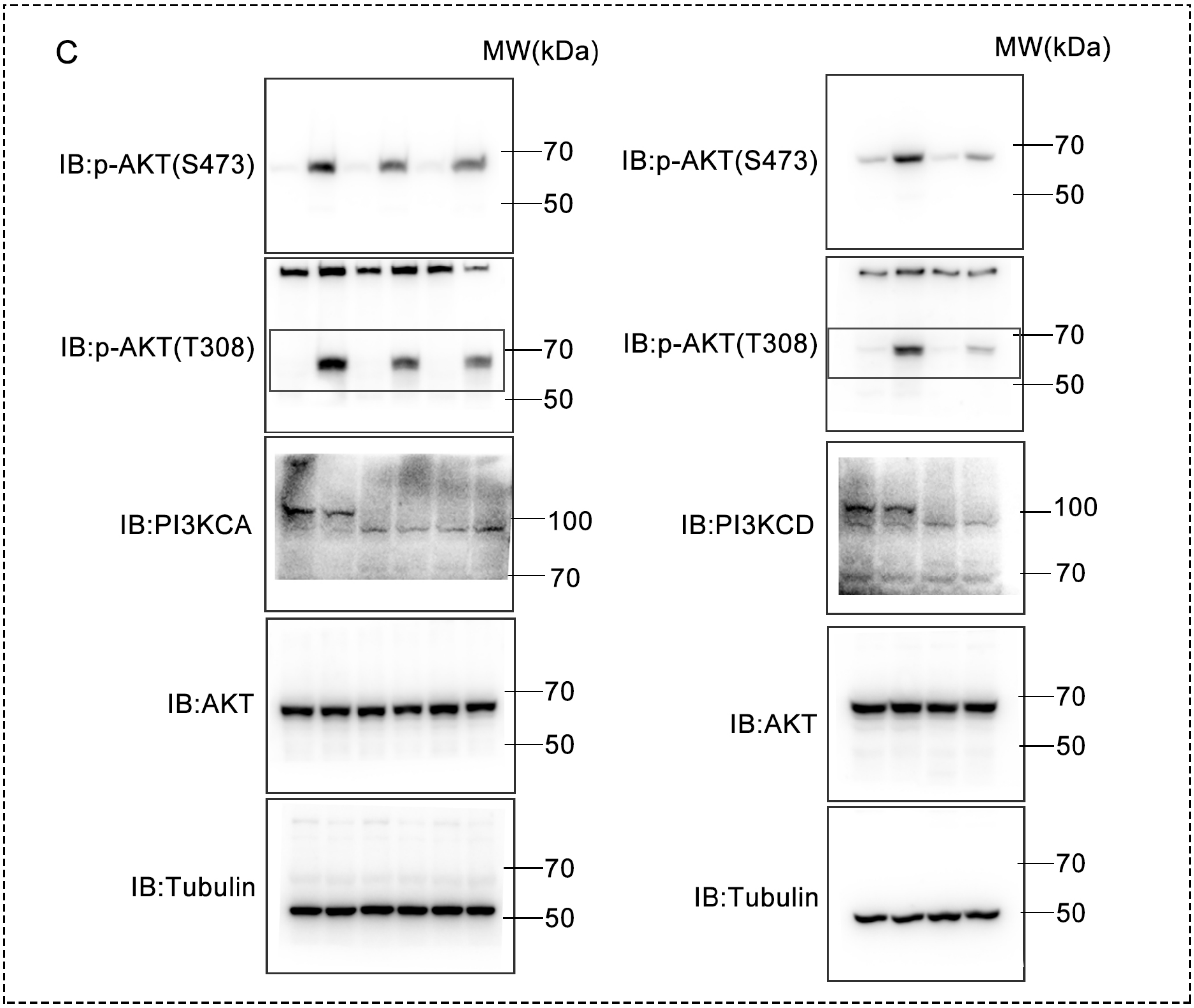

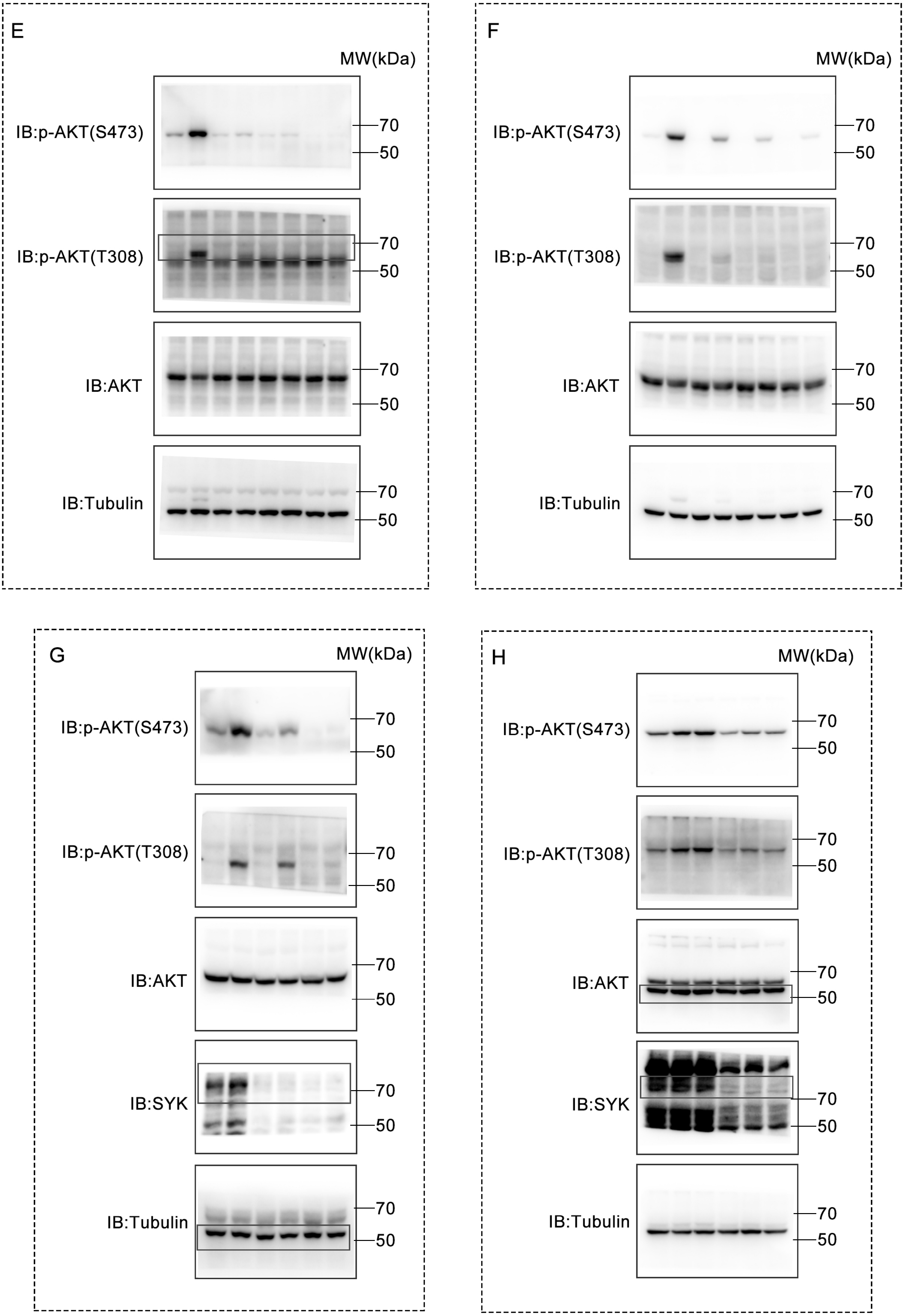
E
MW(kDa)
IB:p-AKT(S473)
IB:p-AKT(T308)
IB:AKT
IB:Tubulin
70
50
F
MW(kDa)
IB:p-AKT(S473)
IB:p-AKT(T308)
IB:AKT
IB:Tubulin
G
MW(kDa)
IB:p-AKT(S473)
IB:p-AKT(T308)
IB:AKT
IB:SYK
IB:Tubulin
H
MW(kDa)
IB:p-AKT(S473)
IB:p-AKT(T308)
IB:AKT
IB:SYK
IB:Tubulin

Uncropped western blot images
Figure S1

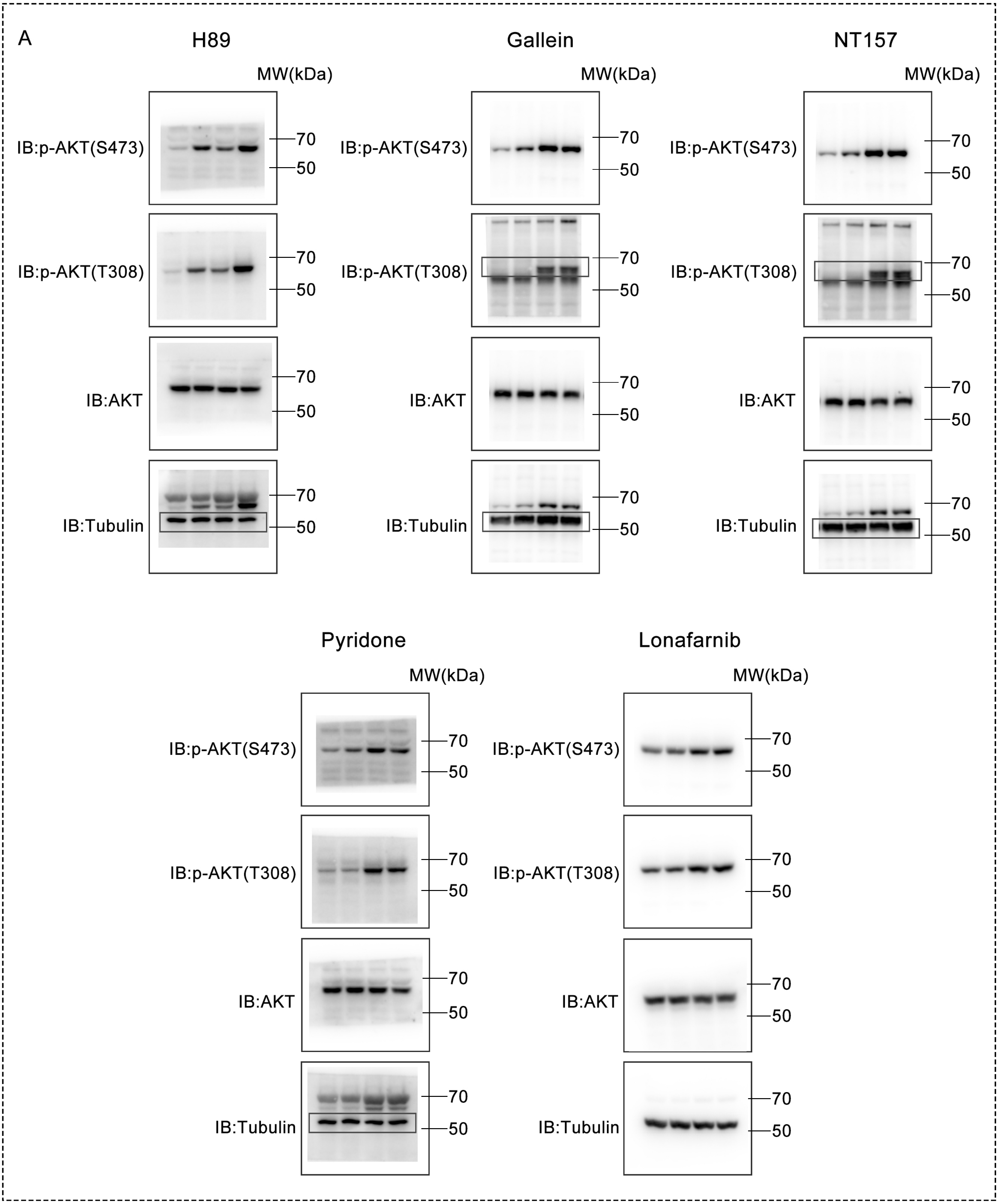

A-2

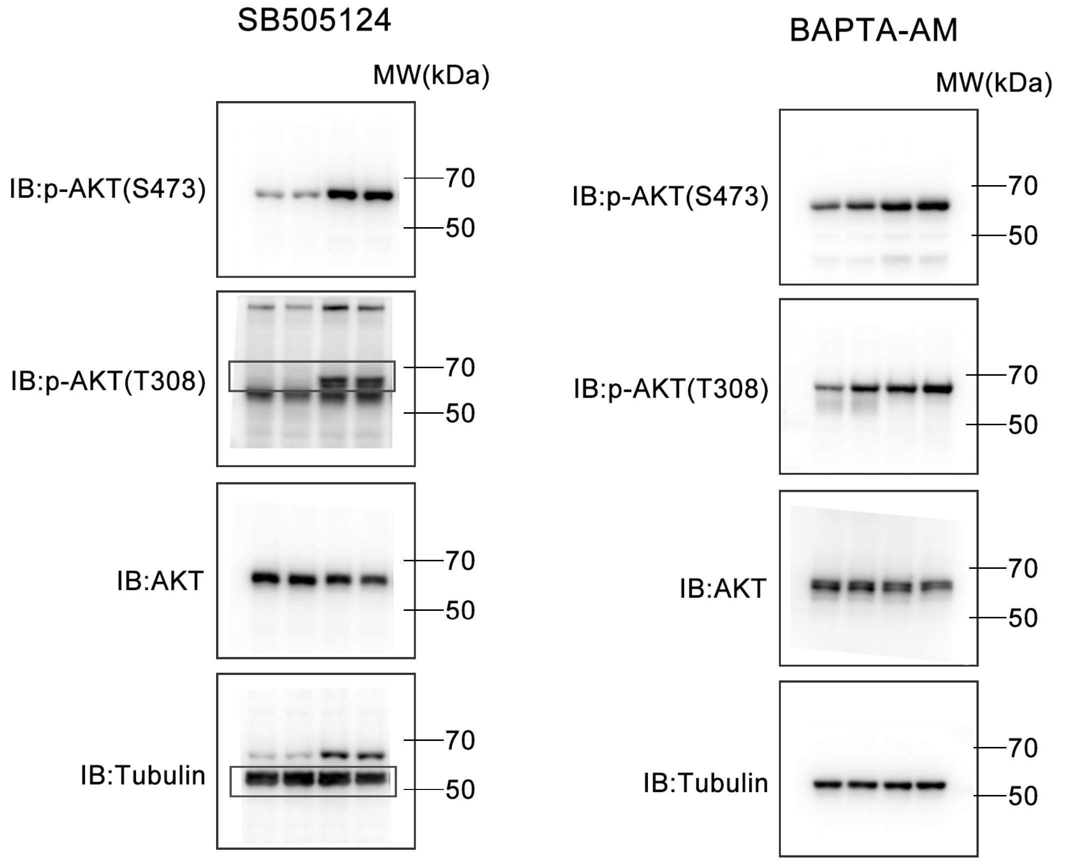

SB505124
BAPTA-AM
MW(kDa)
IB:p-AKT(S473)
IB:p-AKT(T308)
IB:AKT
IB:Tubulin
70
50

B

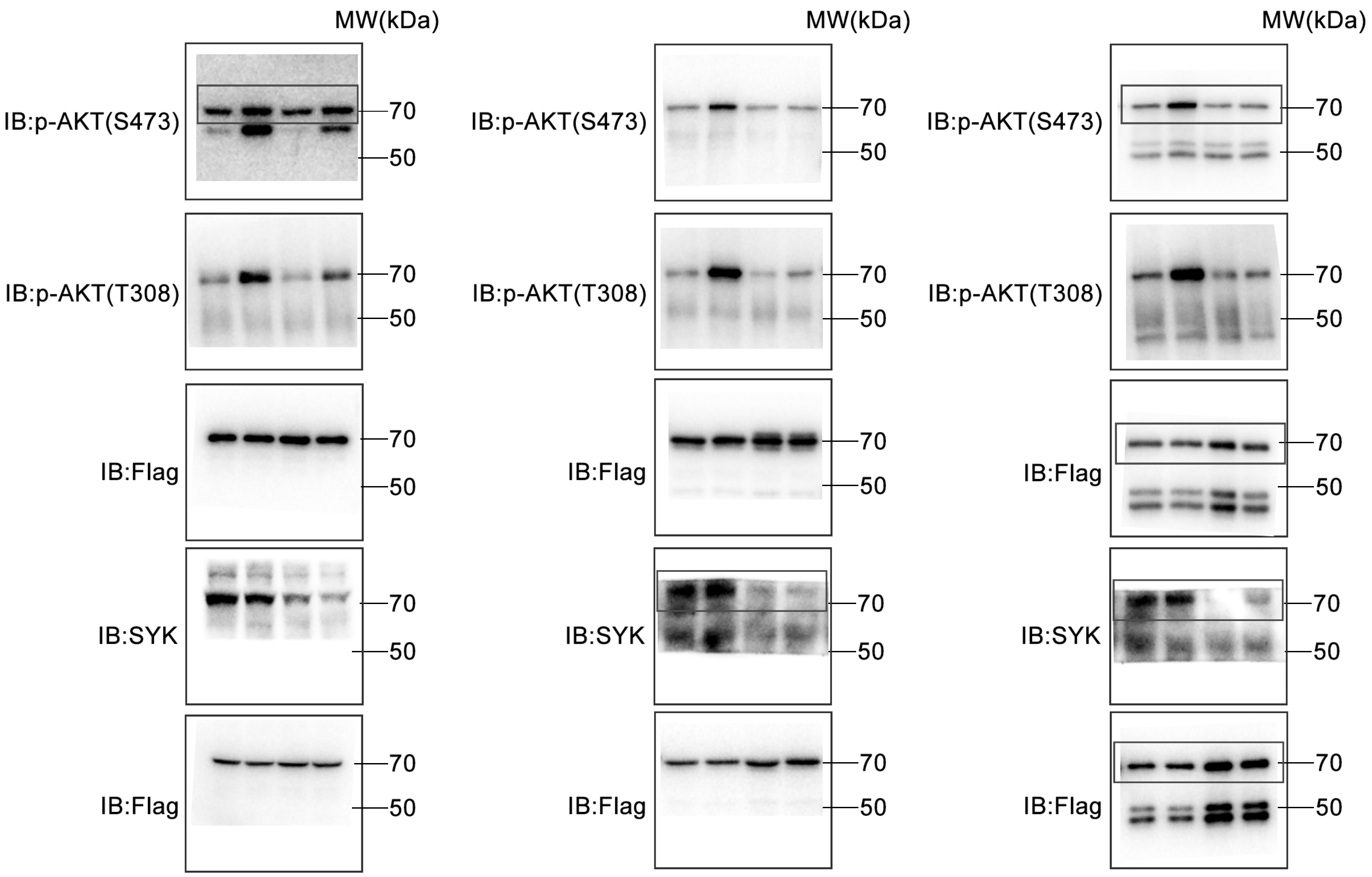

MW(kDa)
IB:p-AKT(S473)
IB:p-AKT(T308)
IB:Flag
IB:SYK
IB:Flag
70
50

Uncropped western blot images
Figure 3

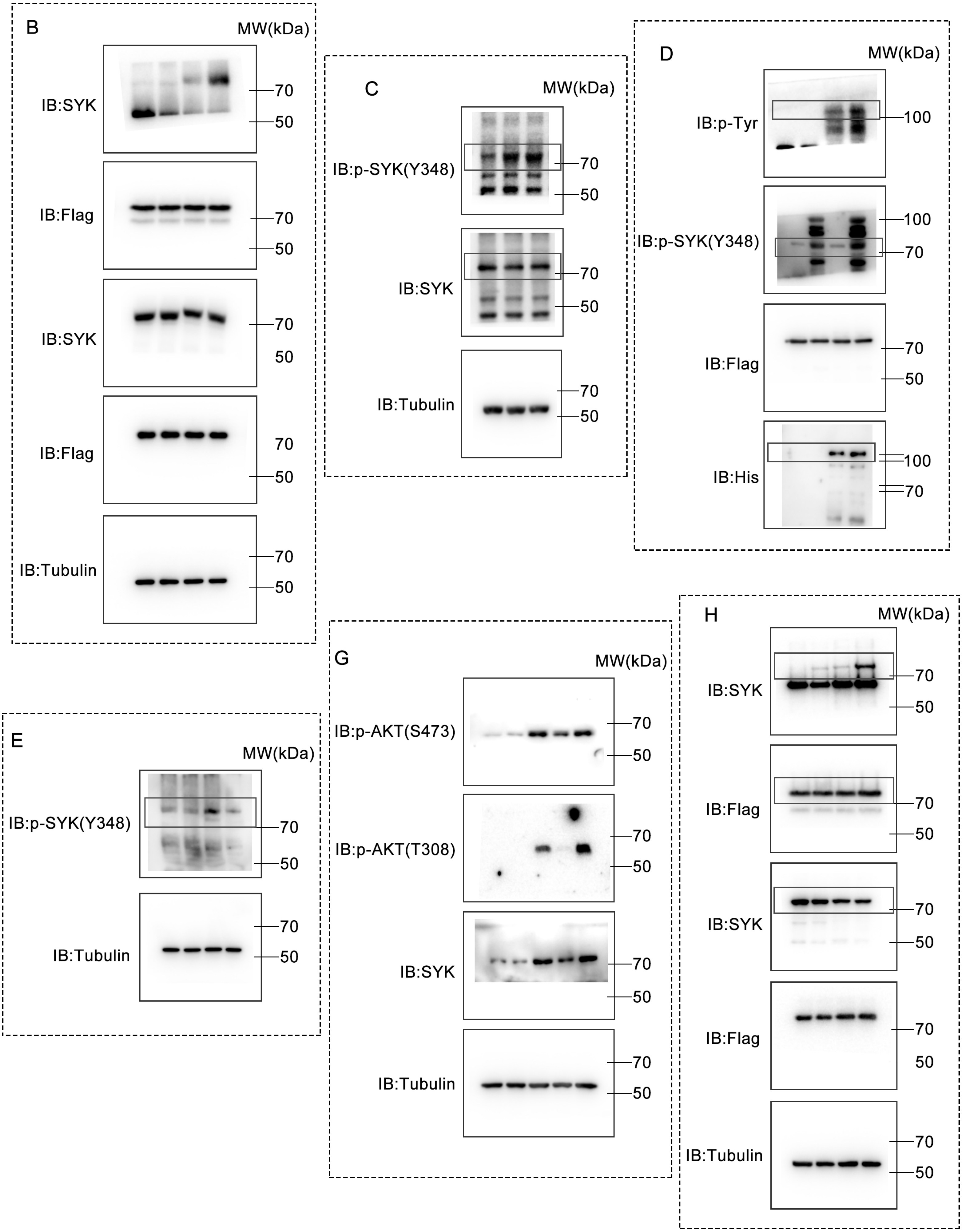

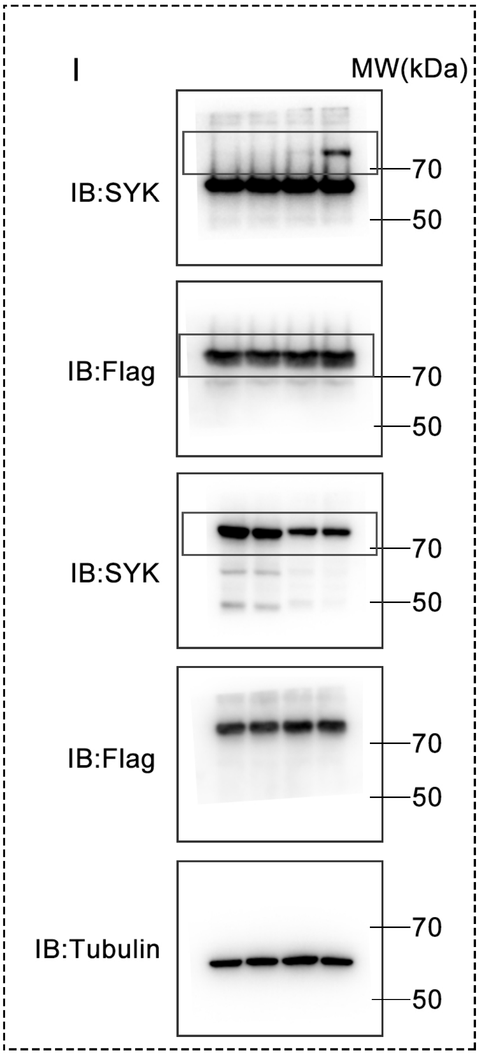
I
MW(kDa)
IB:SYK
IB:Flag
IB:SYK
IB:Flag
IB:Tubulin
70
50

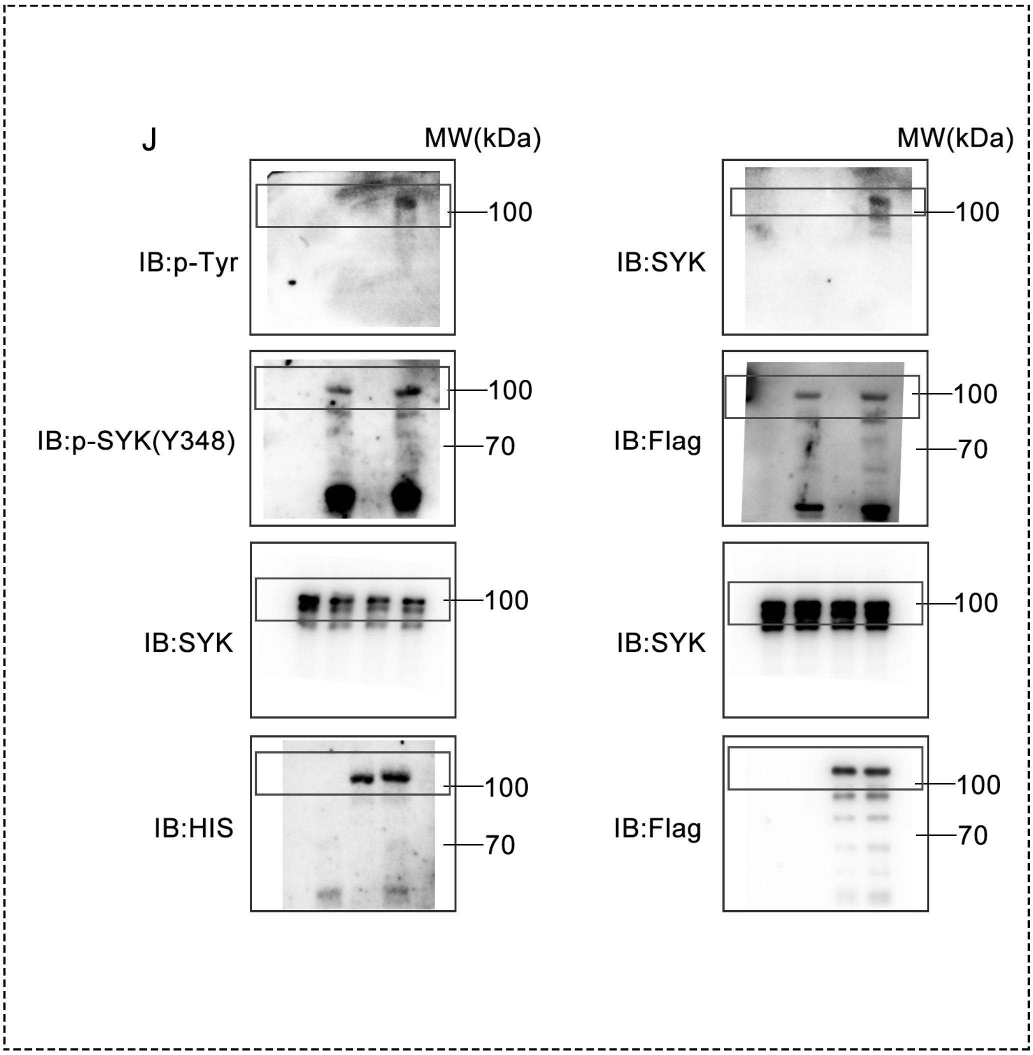
J
MW(kDa)
IB:p-Tyr
IB:p-SYK(Y348)
IB:SYK
IB:HIS
IB:SYK
IB:Flag
IB:SYK
IB:Flag
100
70

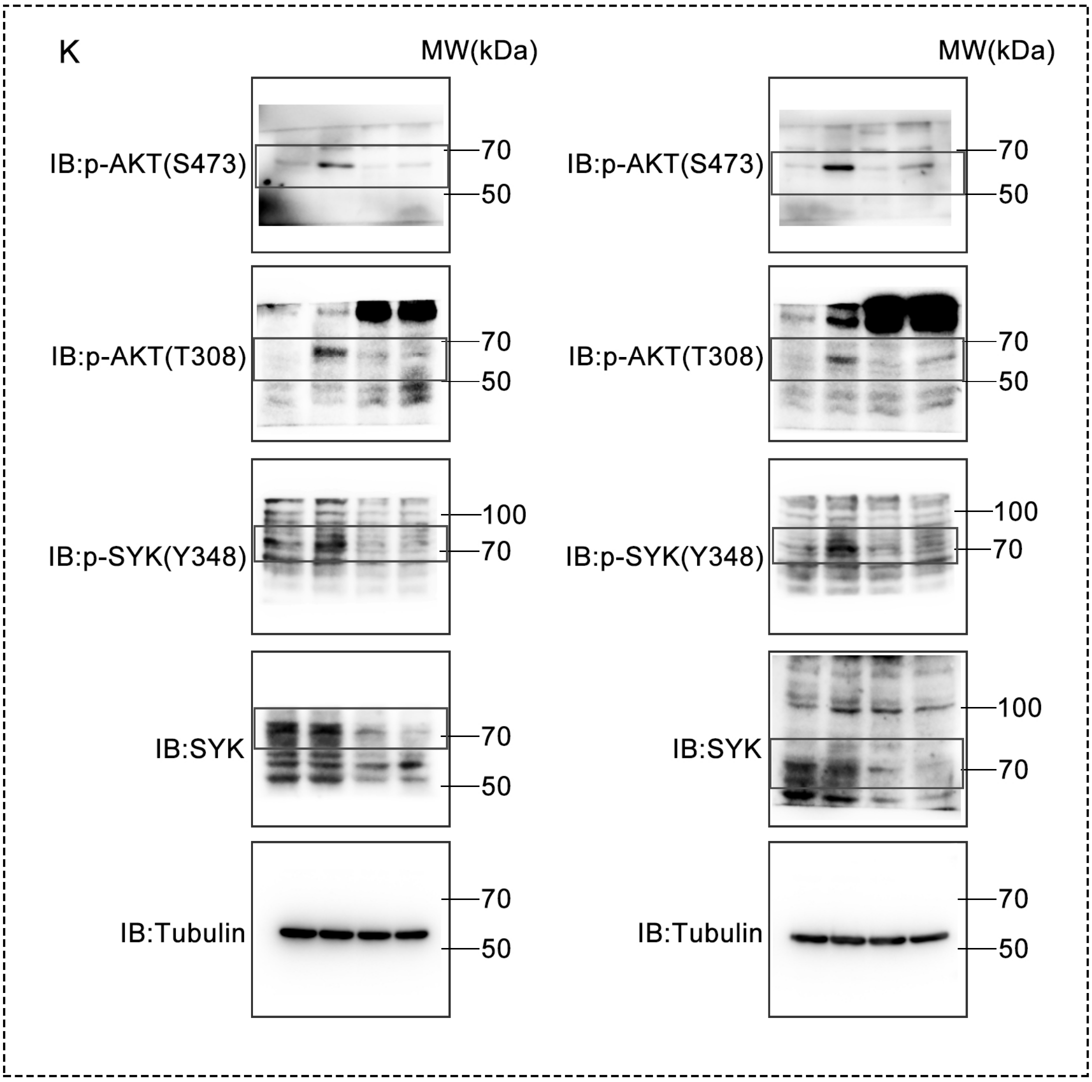
K
MW(kDa)
IB:p-AKT(S473)
IB:p-AKT(T308)
IB:p-SYK(Y348)
IB:SYK
IB:Tubulin
100
70
50

Uncropped western blot images
Figure S2

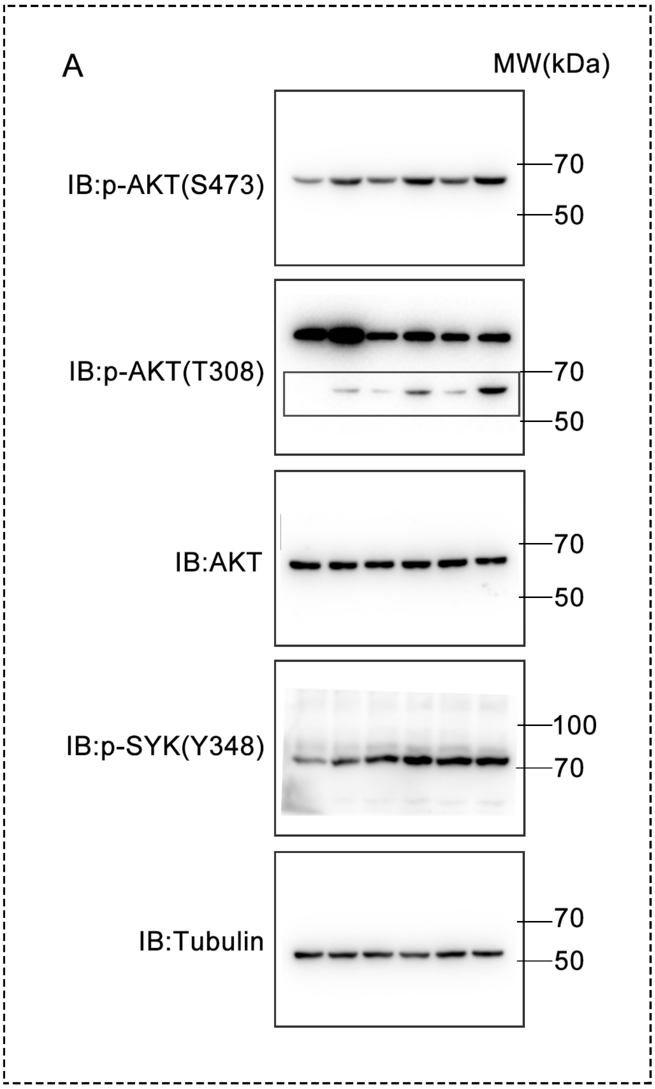

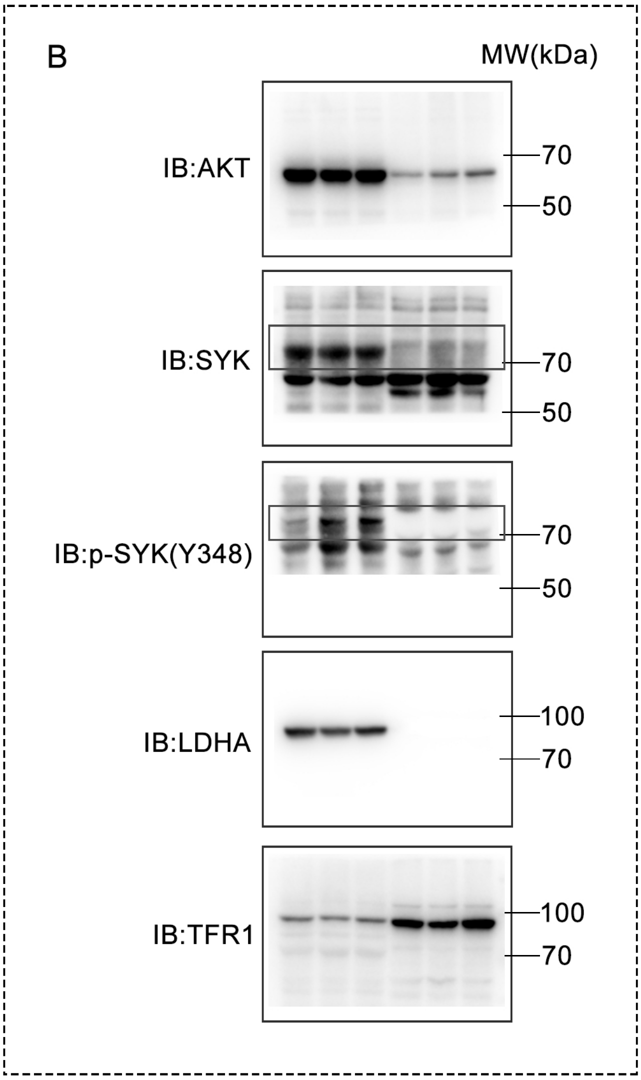

Uncropped western blot images
Figure 4

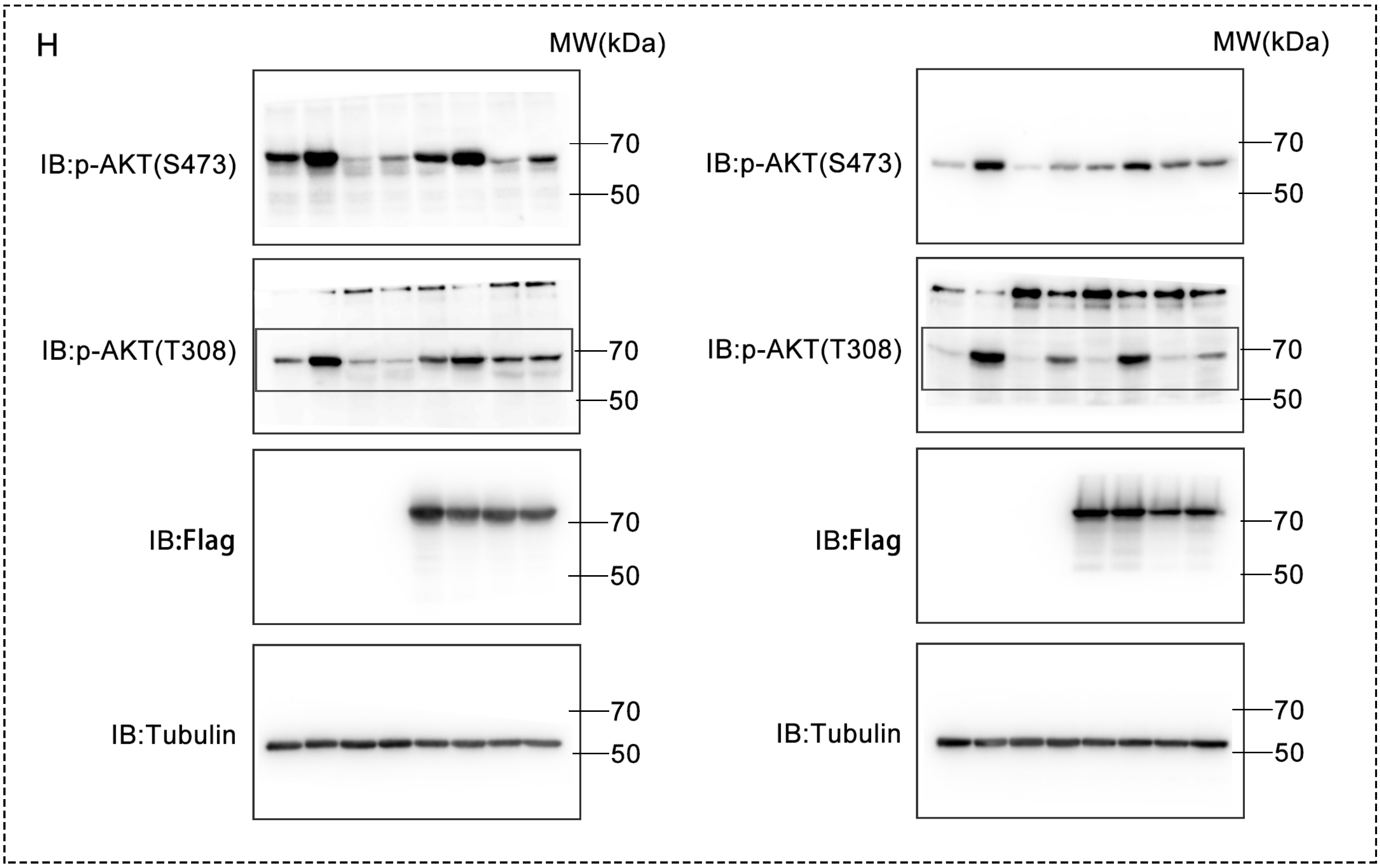

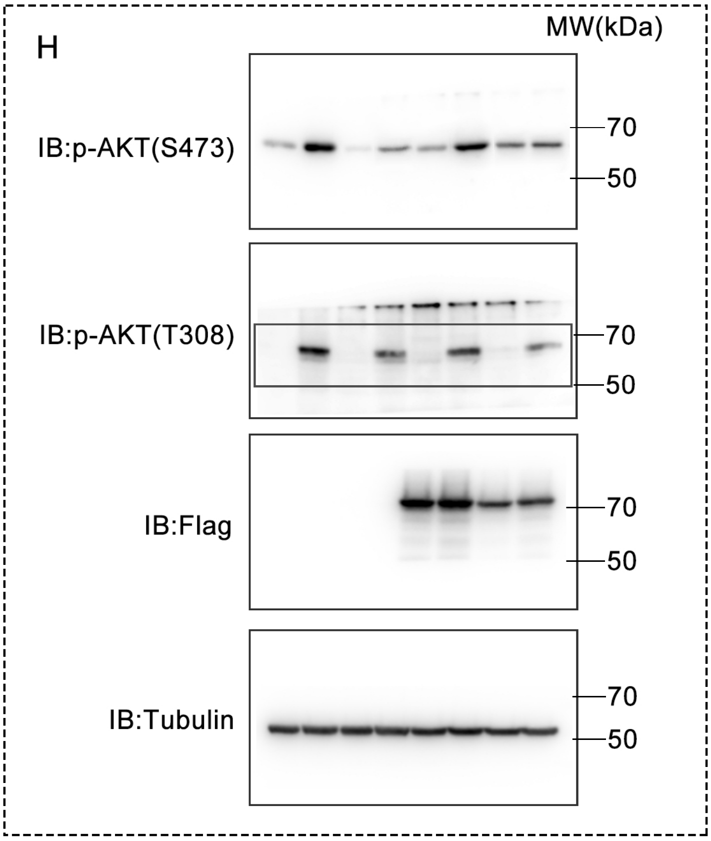

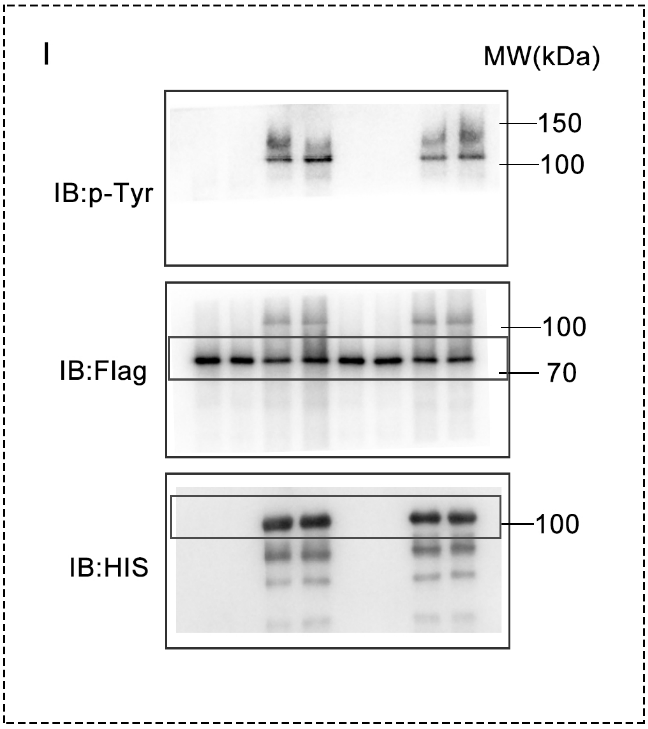

Uncropped western blot images
Figure S3

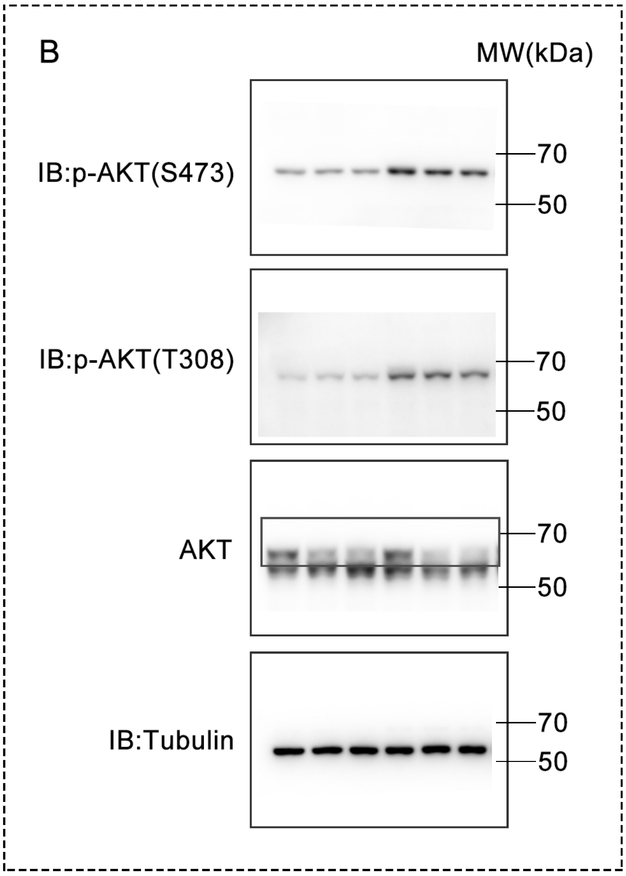

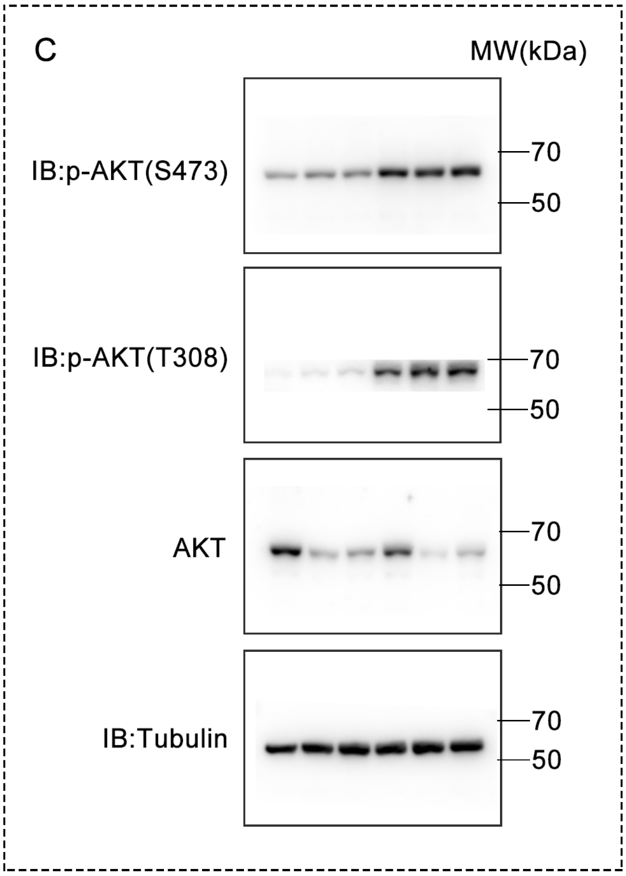

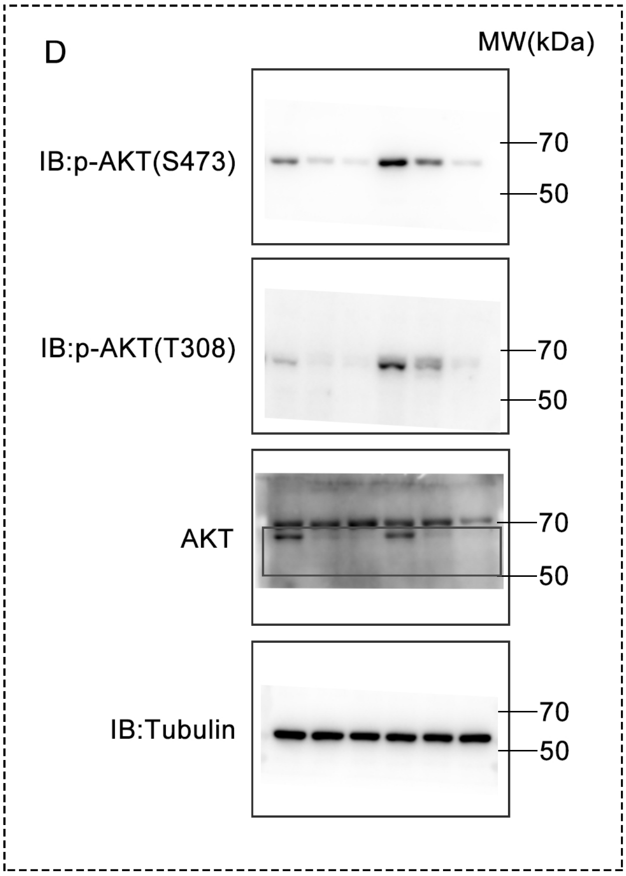

Uncropped western blot images
Figure 6

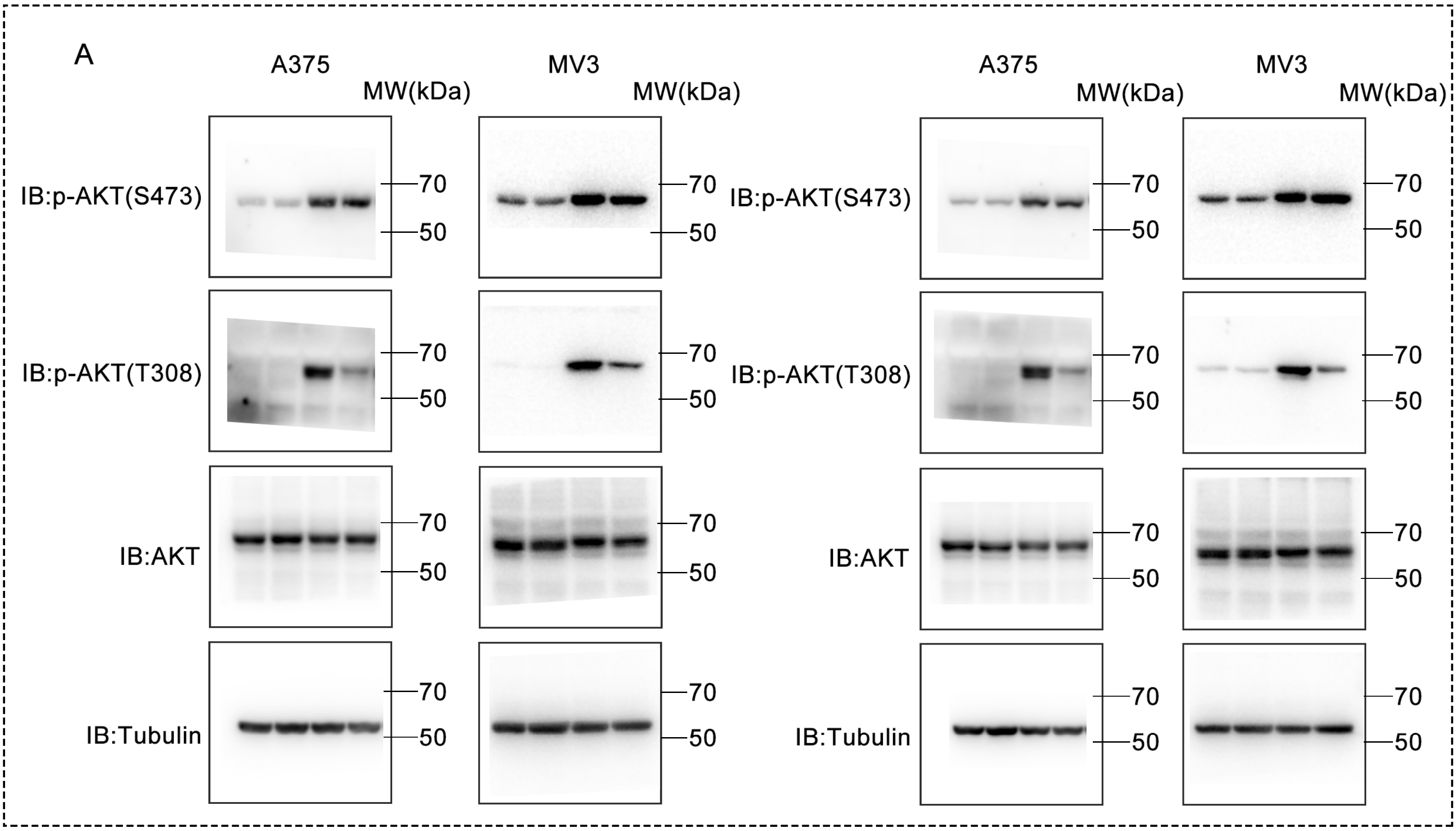

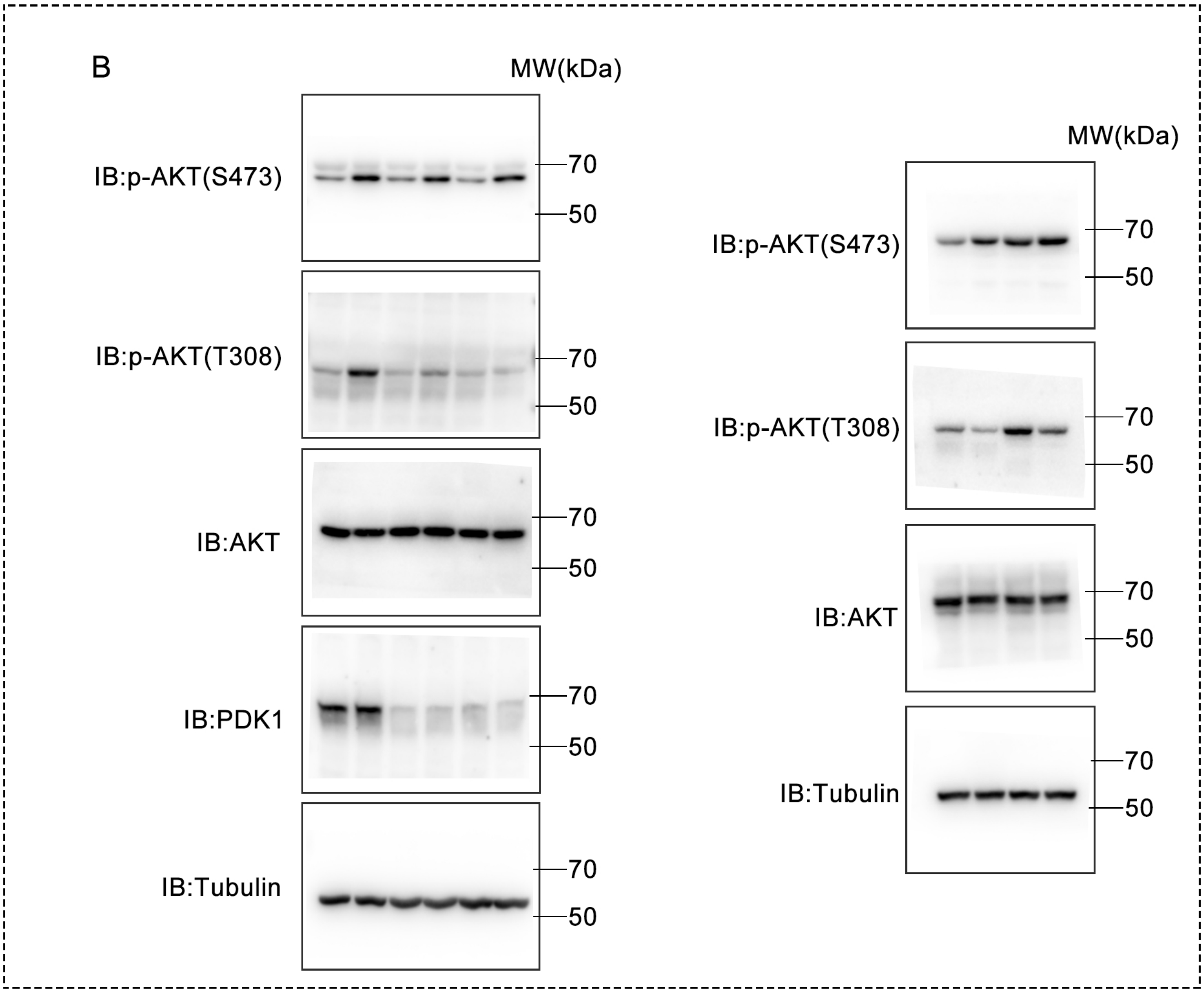

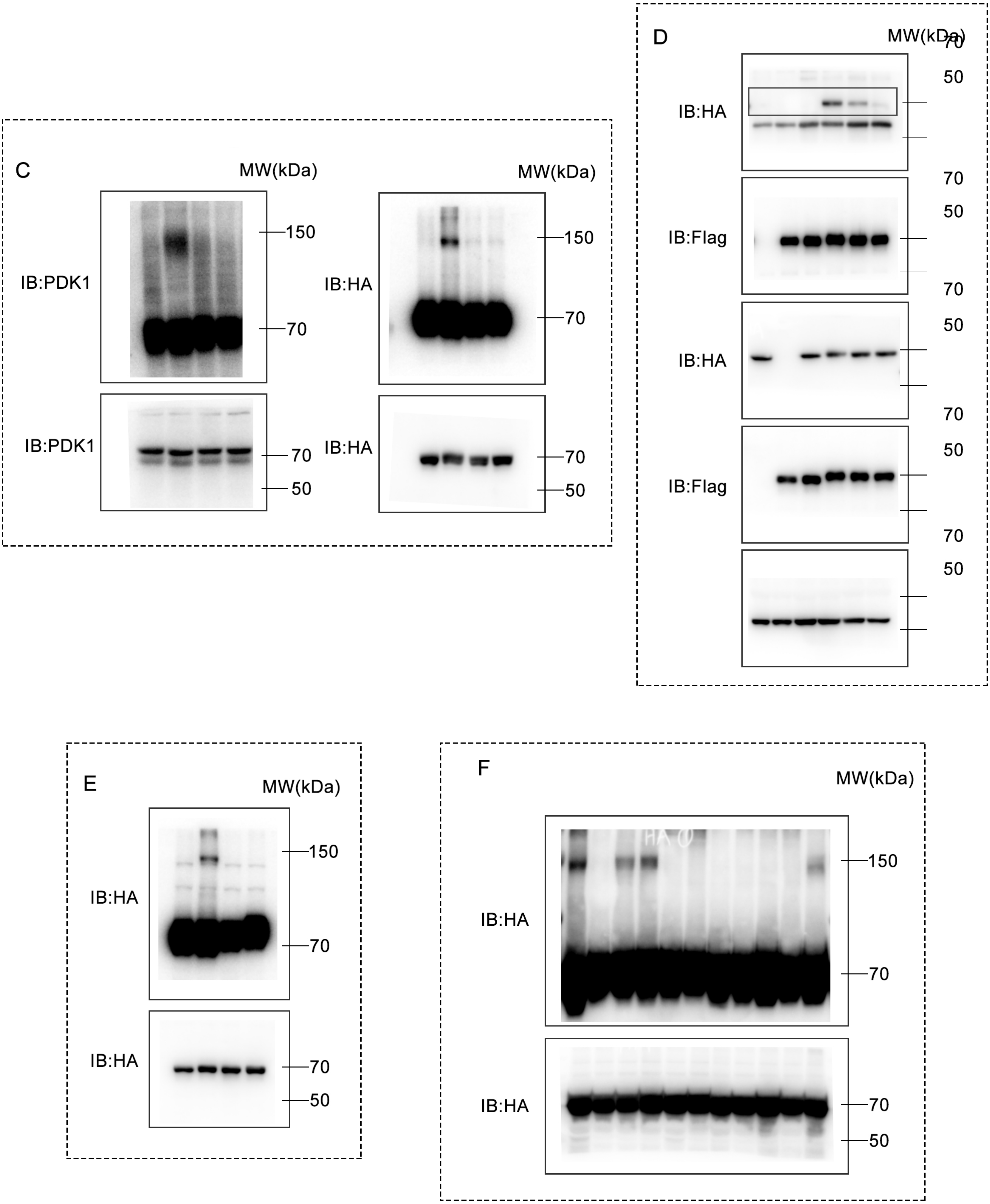
C
MW(kDa)
IB:PDK1
150
70
IB:PDK1
70
50
MW(kDa)
IB:HA
150
70
IB:HA
70
50
D
MW(kDa)
70
50
IB:HA
70
50
IB:Flag
70
50
IB:HA
70
50
IB:Flag
70
50
E
MW(kDa)
IB:HA
150
70
IB:HA
70
50
F
MW(kDa)
IB:HA
150
70
IB:HA
70
50

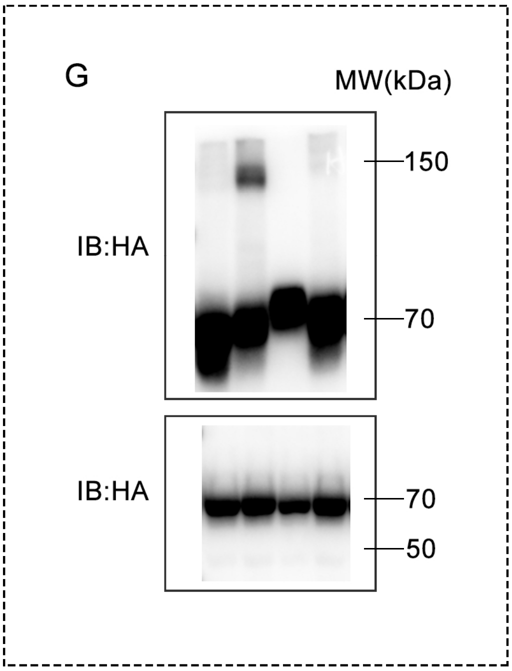
G
MW(kDa)
IB:HA
150
70
IB:HA
70
50

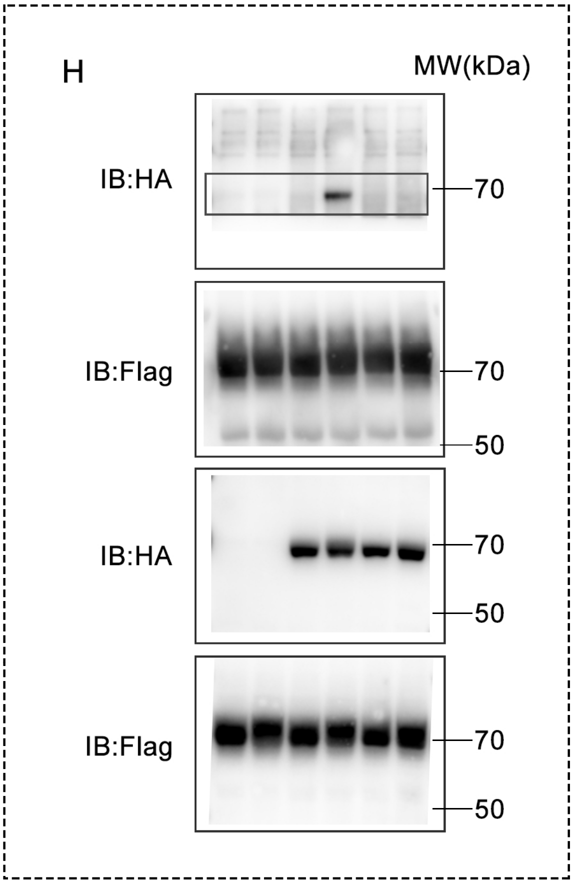
H
MW(kDa)
IB:HA
70
IB:Flag
70
50
IB:HA
70
50
IB:Flag
70
50

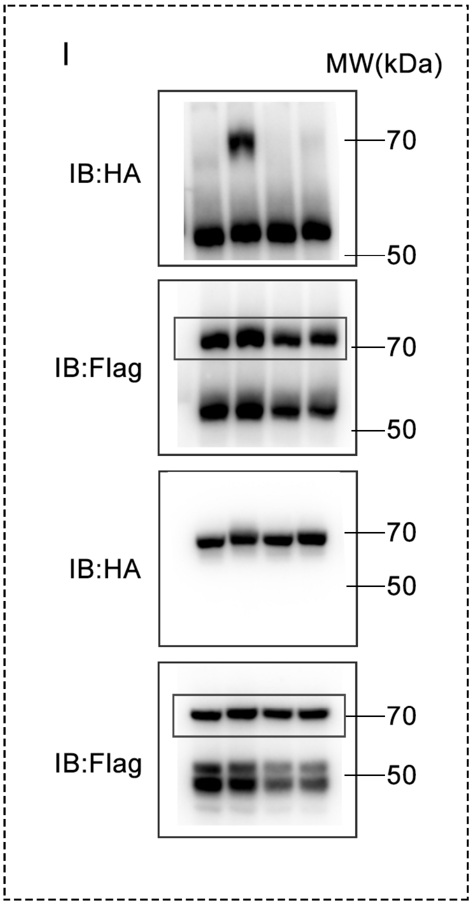
I
MW(kDa)
IB:HA
70
50
IB:Flag
70
50
IB:HA
70
50
IB:Flag
70
50

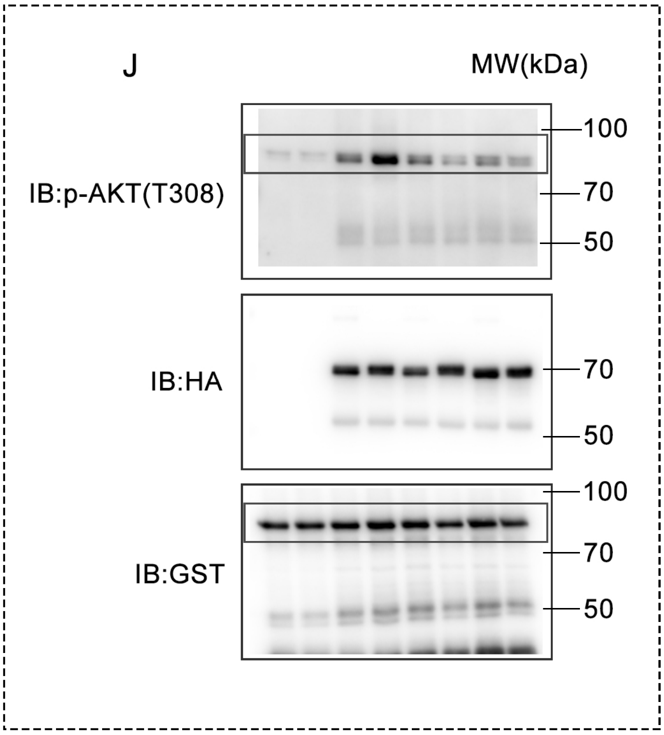
J
MW(kDa)
IB:p-AKT(T308)
100
70
50
IB:HA
70
50
IB:GST
100
70
50

Uncropped western blot images

Figure S4

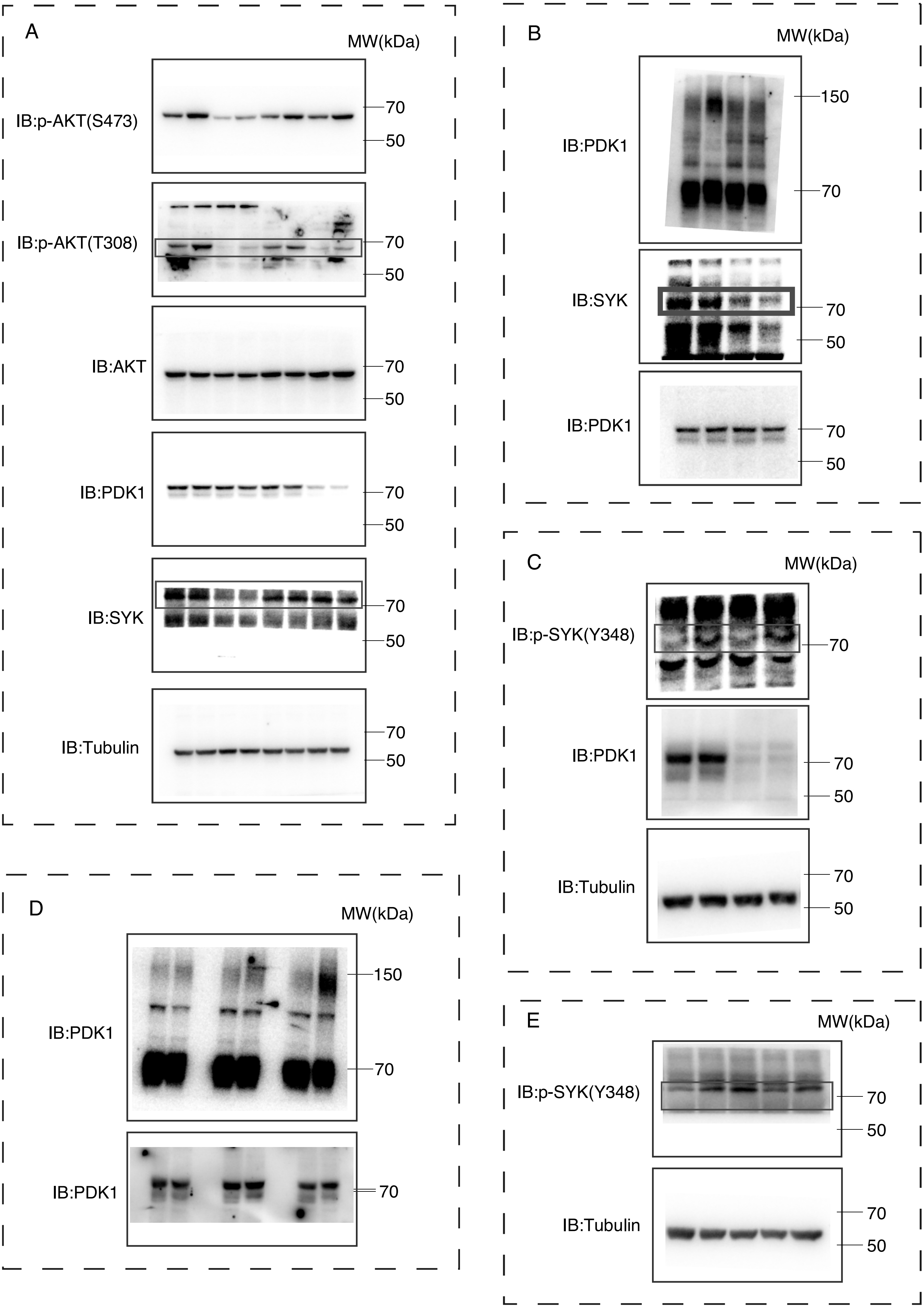

Supplement: Supplementary file 2 — Uncropped Western blot images [file 41419_2025_7906_MOESM2_ESM.pdf]
